# Prognostic Role of Gut Microbiota and Clinical Parameters in Predicting Survival in Advanced Cholangiocarcinoma Patients Receiving Chemotherapy

**DOI:** 10.7150/ijms.131376

**Published:** 2026-06-10

**Authors:** Thanika Ketpueak, Chanon Kunasol, Kanokphong Suparan, Nakarin Inmutto, Sunhawit Junrungsee, Nattayaporn Apaijai, Sasiwan Kerdphoo, Chanisa Thonusin, Chaiyut Charoentum, Thatthan Suksombooncharoen, Sarawut Kongkarnka, Sirinart Kumfu, Lakkhana Sadaow, Wanchai Maleewong, Siriporn C. Chattipakorn, Nipon Chattipakorn

**Affiliations:** 1 Division of Oncology, Department of Internal Medicine, Faculty of Medicine, Chiang Mai University, Chiang Mai, Thailand, 50200.; 2 Neurophysiology Unit, Cardiac Electrophysiology Research and Training Center, Faculty of Medicine, Chiang Mai University, Chiang Mai, Thailand, 50200.; 3 Center of Excellence in Cardiac Electrophysiology Research, Chiang Mai University, Chiang Mai, Thailand, 50200.; 4 Immunology Unit, Department of Microbiology, Faculty of Medicine, Chiang Mai University, Chiang Mai, Thailand, 50200.; 5 Department of Radiology, Faculty of Medicine, Chiang Mai University, Chiang Mai, Thailand, 50200.; 6 Department of Surgery, Faculty of Medicine, Chiang Mai University, Chiang Mai, Thailand, 50200.; 7 Cardiac Electrophysiology Unit, Department of Physiology, Faculty of Medicine, Chiang Mai University, Chiang Mai, Thailand, 50200.; 8 Department of Pathology, Faculty of Medicine, Chiang Mai University, Chiang Mai, Thailand, 50200.; 9 Mekong Health Science Research Institute, Khon Kaen University, Khon Kaen, Thailand, 40002.; 10 Department of Parasitology, Faculty of Medicine, Khon Kaen University, Khon Kaen, Thailand, 40002.; 11 Department of Oral Biology and Diagnostic Sciences, Faculty of Dentistry, Chiang Mai University, Chiang Mai, Thailand, 50200.

**Keywords:** gut microbiota, cholangiocarcinoma, hepatocellular carcinoma, *Opisthorchis viverrini*, survival

## Abstract

**Introduction:**

Cholangiocarcinoma (CCA) is high prevalent in Asia. Emerging evidence indicates gut and tissue microbiota are involved in CCA carcinogenesis and progression. This study investigated the potential role of gut and tissue microbiota as prognostic biomarkers associated with treatment outcomes in advanced intrahepatic CCA (ICCA) patients.

**Methods:**

This prospective study comprised two cohorts: hepatocellular carcinoma (HCC) or ICCA patients undergoing surgery or biopsy, and unresectable ICCA patients treated with cisplatin and gemcitabine. Gut bacterial profiles were characterized from pre-treatment fecal samples using 16S rRNA and analyzed with clinical outcomes. Tissue bacterial profiles were determined by real-time qPCR.

**Results:**

In 30 ICCA patients receiving chemotherapy, gut microbiota differed based on chemotherapy response, PFS, overall survival, and *Opisthorchis viverrini* infection. An increase in *Acidaminococcus* and *Sutterella*, along with a decrease in NK4A214_group, Lachnospiraceae_FCS020_group, and UCG-010, were associated with poor clinical outcomes. Notably, *Intestinimonas* levels were significantly associated with less progression of disease within six months (aOR 0.92). Of total 70 patients, bacterial profiles of ICCA and HCC tissues did not significantly differ.

**Conclusion:**

Advanced ICCA patients with poor outcomes following chemotherapy exhibited the distinct pattern of gut microbiota, which might be used as potential prognostic biomarkers.

## Introduction

Cholangiocarcinoma (CCA) is a rare cancer globally but has a notably higher prevalence in Asia. From 2000 to 2007, CCA incidence in Europe was 2.4 per 100,000 per year, whereas the Northeast Thailand reported 80 per 100,000 per year 1. Risk factors associated with CCA include hepatitis B or C virus infection, primary biliary sclerosis, diabetes, and recurrent biliary tract infection. Notably, liver fluke infection, particularly *Opisthorchis viverrini* (OV) 2, is a key risk factor and classified as a group one carcinogen by the International Agency for Research on Cancer (IARC).

To date, gut microbiota, the resident microorganisms living inside the host gastrointestinal tract, has been elucidated in malignancies such as colorectal cancer (CRC) 3. However, the role of gut and tissue-specific microbiota in CCA remains poorly investigated 4. Recent evidence indicates that OV-infected CCA and non-OV-infected CCA differ in tissue-specific microbiota, with OV altering liver microbiota and producing toxic metabolites that drive disease progression 5. Clinical studies further showed that CCA exhibited the highest microbiota biodiversity compared to hepatocellular carcinoma (HCC), liver cirrhosis, and normal controls 6. Notably,* Ruminococcaceae* species were linked to the vascular invasion of ICCA 6. Additionally, elevated levels of encapsulated bacteria, such as *Klebsiella pneumoniae,* correlated with poorer survival in CCA patients 7. These findings suggest that the host's microbiota profile may play a role in CCA tumorigenesis and prognosis. However, the relationship between gut and cancer tissue-specific microbiota remains unclear. Prior studies reported variations in microbiota across specimens, with general increase in *Helicobacter* spp., *Bifidobacteriaceae*, and *Ruminococcaceae* in CCA patients, with *Bifidobacteriaceae* and *Enterobacteriaceae* elevated in OV-infected CCA tissues 5**.** Standard first-line therapy with cisplatin and gemcitabine provides limited survival benefits 8, and no established predictive biomarkers for chemotherapy responsiveness or prognosis. This study aimed to investigate whether tissue-specific and gut microbiota profiles could serve as novel prognostic biomarkers for CCA patient outcomes. Addressing this gap may facilitate personalized therapeutic strategies, integrating microbiota as a potential prognostic tool.

## Methods

### Study design and participants

This prospective study comprised two cohorts. Eligible participants were required to be aged ≥18 years, have an Eastern Cooperative Oncology Group (ECOG) performance status of 0-1, and have adequate hematologic and biochemical function. For the first cohort, patients with metastatic, unresectable, or recurrent clinically suspected intrahepatic cholangiocarcinoma (ICCA) who were planned for diagnostic biopsy, as well as patients with early-stage ICCA or hepatocellular carcinoma (HCC) scheduled for surgical resection, were recruited. Patients who refused tissue diagnosis or surgery, or who were diagnosed with liver metastases from other primary cancers, were excluded. Patients with advanced ICCA—defined as histologically confirmed metastatic, unresectable, or recurrent ICCA—were eligible for inclusion in the second cohort. These patients were planned to receive first-line low-dose cisplatin and gemcitabine at Maharaj Nakorn Chiang Mai Hospital, Chiang Mai, Thailand, between April 1, 2021, and September 30, 2023.

Treatment followed a standard protocol: cisplatin 25 mg/m^2^ D1, D8 and gemcitabine 1,000 mg/m^2^ D1, D8 every 21 days cycle up to eight cycles 8. Following cycles four and eight, the patient was appointed for response evaluation using a computed tomography (CT) scan. This study was approved by the Ethics Committee of the Faculty of Medicine, Chiang Mai University, Thailand, following the Declaration of Helsinki and Good Clinical Practice Guidelines (Approval Number: MED-2563-07684). All participants signed the informed consent.

### Outcomes

**Figure 1:**
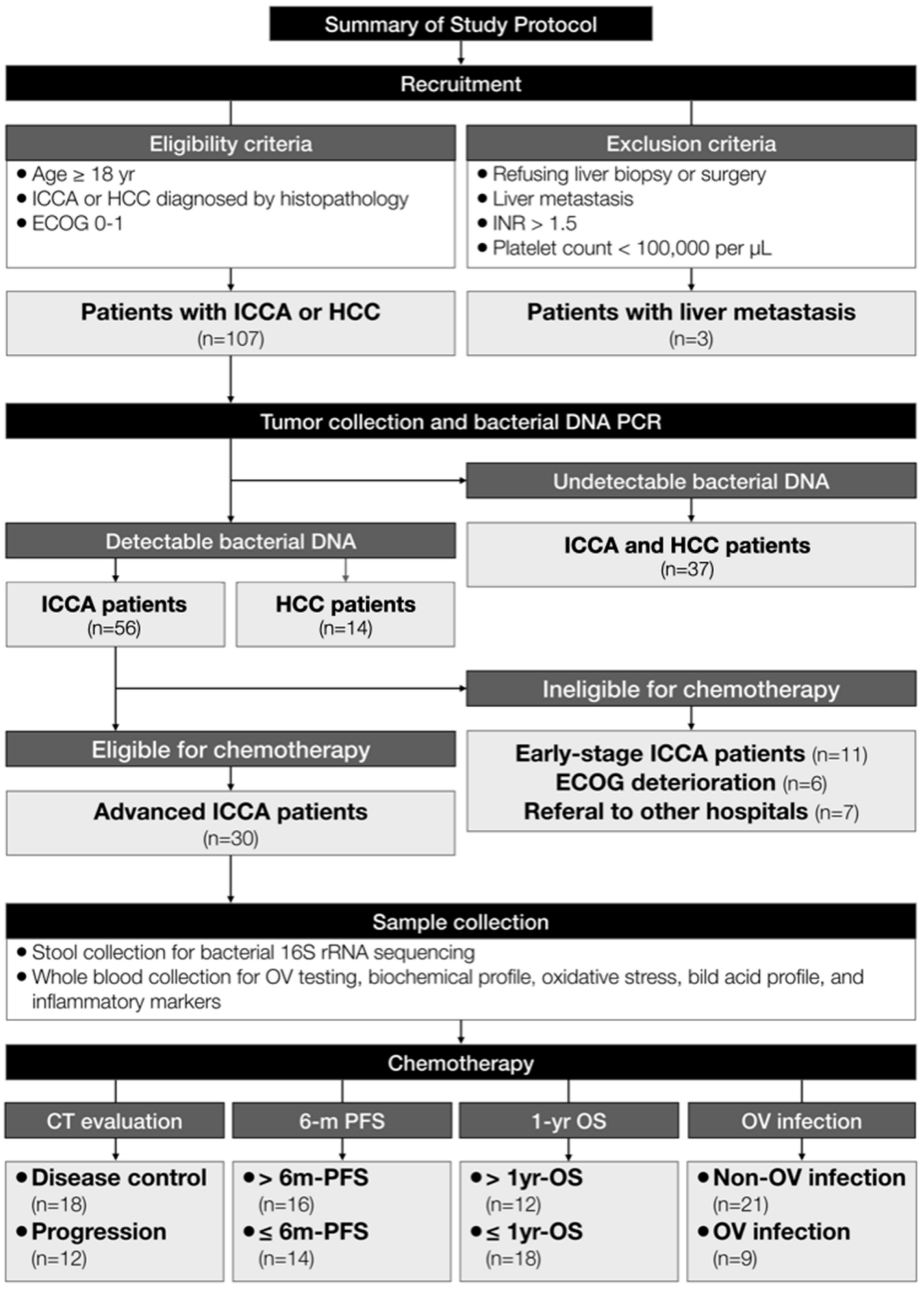
The study protocol demonstrated eligibility and exclusion criteria. Of 120 patients, liver cancer tissue was collected from 107 patients to evaluate microbiota by RT-qPCR. After that, advanced ICCA patients who received chemotherapy were recruited into Cohort 2 with evaluation of stool microbiota as well as blood biochemical profiles. **Abbreviation:** 16S rRNA, 16s ribosomal ribonucleic acid; CT, computed tomography, DNA, deoxyribonucleic acid; ECOG, Eastern Cooperative Oncology Group; ICCA, intrahepatic cholangiocarcinoma; INR, international normalized ratio; HCC, hepatocellular carcinoma; OS, overall survival; OV, Opisthorchis viverrini; PCR, polymerase chain reaction PFS, progression-free survival.

The primary outcome for gut microbiota was to evaluate the correlation between gut bacterial profiles and clinical outcomes in advanced ICCA patients, including response to chemotherapy, progression-free survival (PFS), and overall survival (OS). The secondary outcomes were to determine gut microbiota profiles among patients with OV-infected ICCA and to analyze other prognostic factors associated with poor survival - defined by 1) ICCA progression evaluated by CT scan after Cycle 4 of chemotherapy, 2) ICCA progression within six months (≤ 6m-PFS), and 3) death within one year (≤ 1yr-OS). The exploratory outcome for tissue microbiota was to identify the differences of bacterial profiles between ICCA and HCC tissues. The study protocol was summarized in **Figure 1**. Details of the laboratory processes and tests were described in the **Supplementary Materials**.

### Statistical analysis

Statistical analyses were performed using R (v.4.3.1). Clinical data were expressed as median with IQR, mean ± SEM, or numbers with percentages. The Shapiro-Wilk test was employed to determine the normality of numerical data. Corresponding statistical tests - including the Kruskal-Wallis test with Dunn's post hoc test or the Mann-Whitney U test for nonparametric data, one-way ANOVA with Tukey's post hoc test for parametric data, and the simulated chi-square test for categorical data - were performed. A p-value < 0.05 was considered statistically significant.

Univariate analyses were first performed to identify variables significantly associated with the outcomes. Variables showing statistical significance were subsequently considered for inclusion in the multivariable logistic regression models. Final models were constructed by selecting a limited number of predictors based on both statistical significance and clinical relevance, while minimizing the risk of overfitting given the limited sample size. No automated variable selection methods were applied. Multivariable logistic regression models were used to evaluate the association between clinical variables, gut microbiota taxa, and clinical outcomes. Adjusted odds ratios (aOR) with 95% confidence intervals (CI) were calculated.

## Results

### Tissue microbial profiles between ICCA and HCC did not differ

A total of 110 patients were prospectively enrolled in this study. Thirty-seven patients were excluded because bacterial targets in the cancerous tissues did not produce detectable amplification during RT-qPCR. In addition, three patients with liver metastases from colorectal cancer were excluded from the analysis. Of the remaining 70 patients, 56 patients were diagnosed with ICCA and 14 patients had HCC. Baseline clinical characteristics (**Supplementary Table 1**) showed no age differences between the two groups, while proportions of sex and body weight were different. Cirrhosis, HBV, and HCV infection were more prevalent in HCC. Other risk factors, including smoking, alcohol, and raw food consumption, showed no significant differences between the two groups. The majority of ICCA patients had metastatic disease (83.0%), while HCC patients had non-metastatic disease (85.7%). However, no significant differences were observed in the abundance of cancerous tissue bacteria, including *Eubacteria*, *Clostridiales*, *Lactobacillus*, *Bacteroides*, and *Enterobacteriaceae*, or in the Firmicutes-to-Bacteroidota (F/B) ratio between the ICCA and HCC groups (Supplementary Table 2). After adjustment for major clinical factors, including cirrhosis status, HBV infection, HCV infection, and disease stage (metastatic vs non-metastatic), the results remained unchanged, with no significant differences observed in tissue bacterial abundance between ICCA and HCC. Furthermore, no significant association was observed between tissue bacterial quantification assessed by RT-qPCR and gut microbiota profiles determined by 16S rRNA sequencing (**Supplementary Table 2**). Among ICCA patients, the abundance of cancerous tissue bacteria did not differ between those with poor and good clinical outcomes (**Supplementary Table 3**).

### Progression-free survival was different in advanced ICCA patients depending on chemotherapy response and OV infection

Thirty patients with advanced ICCA, who were available for the chemotherapy, were further recruited in the second cohort. The baseline characteristics of these patients were divided into two groups using three indicators determining clinical outcomes: 6m-PFS, 1yr-OS, and status of OV infection, as listed in **Figure 1** and **Table 1**. Age, sex, risk factors, and staging were not significantly different between the two groups. For 6m-PFS, the prevalence of OV positive patients was significantly higher in the ≤ 6m-PFS group (50.0%) compared to the > 6m-PFS group (12.5%). Most of the patients in the ≤ 6m-PFS group, 85.7% had progression of disease from response evaluation using CT scan. For 1yr-OS, only objective response rate (ORR) was different between the two groups, with higher disease progression in the ≤ 1yr-OS group (66.7%) compared to the > 1yr-OS group (0.0%). Next, no differences in baseline characteristics were observed between the OV infected and non-OV infected groups.

**Figure 2:**
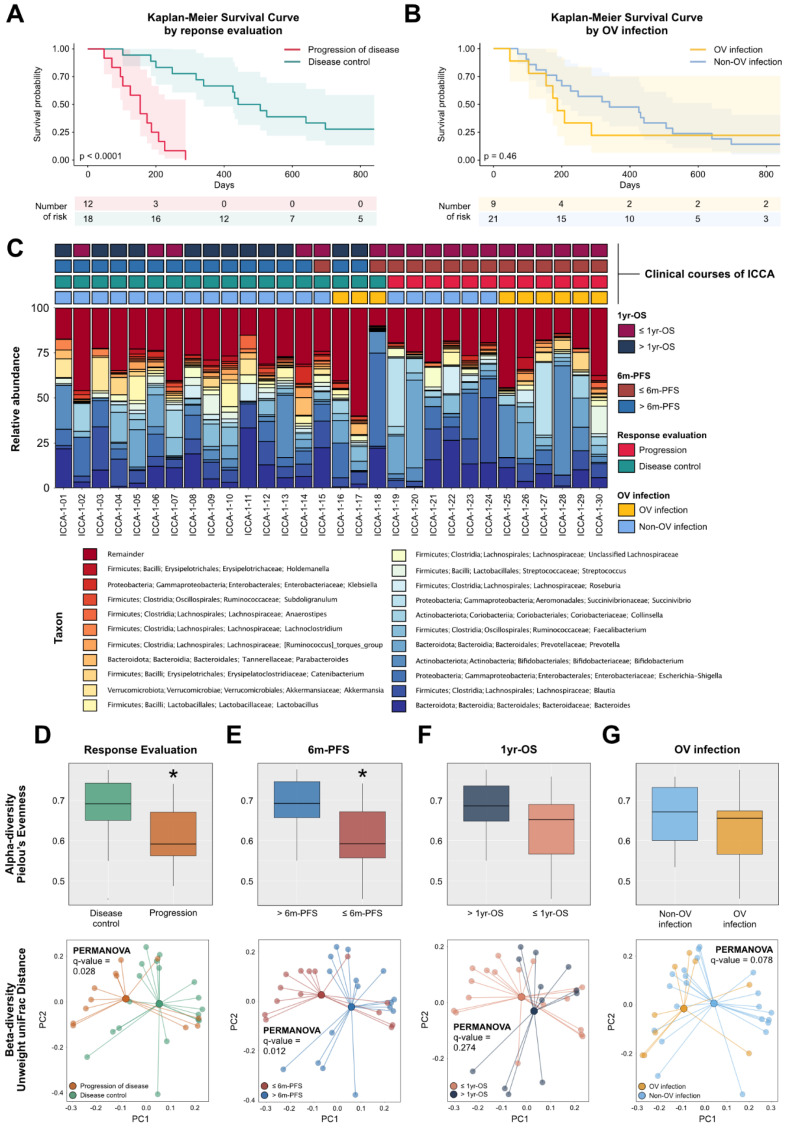
** (A)** Kaplan-Meier graph demonstrating PFS categorized by CT evaluation. **(B)** Kaplan-Meier graph demonstrating PFS categorized by OV infection status. **(C)** Taxonomic bar plots illustrating relative abundance of gut microbial composition of each subject. **(D)** Alpha- and beta-diversity indices showing bacterial alteration categorized by response evaluation. **(E)** Alpha- and beta-diversity indices showing bacterial alteration categorized by 6m-PFS. **(F)** Alpha- and beta-diversity indices showing bacterial alteration categorized by 1yr-OS. **(G)** Alpha- and beta-diversity indices showing bacterial alteration categorized by OV infection. **Abbreviation:** ICCA, intrahepatic cholangiocarcinoma; OS, overall survival; OV, Opisthorchis viverrini; PC, principal component; PCR, polymerase chain reaction PFS, progression-free survival.

Evaluating chemotherapy responses in Cohort two by using CT evaluation, four patients (13.3%) had partial response, 14 patients had (46.7%) stable disease, and 12 (40.0%) patients had progression of disease as the best response. The ORR was 13.3%, disease control rate (DCR) was 60.0%, and 40.0% of patients experienced a progression of disease (PD). The median PFS (mPFS) was 8.56 months in the DCR group and 2.0 months in the PD group, with HR 26.9 (95%CI 5.72-126.5; p-value < 0.001) (**Figure 2A**). In addition, the mPFS was 7.30 months in the non-OV infected group and 3.38 months in the OV infected group, with HR 1.57 (95% CI 0.70-3.51; p-value 0.27) (**Figure 2B**).

### Gut microbiota profiles among chemotherapy responsive and non-responsive patients

**Figure 3:**
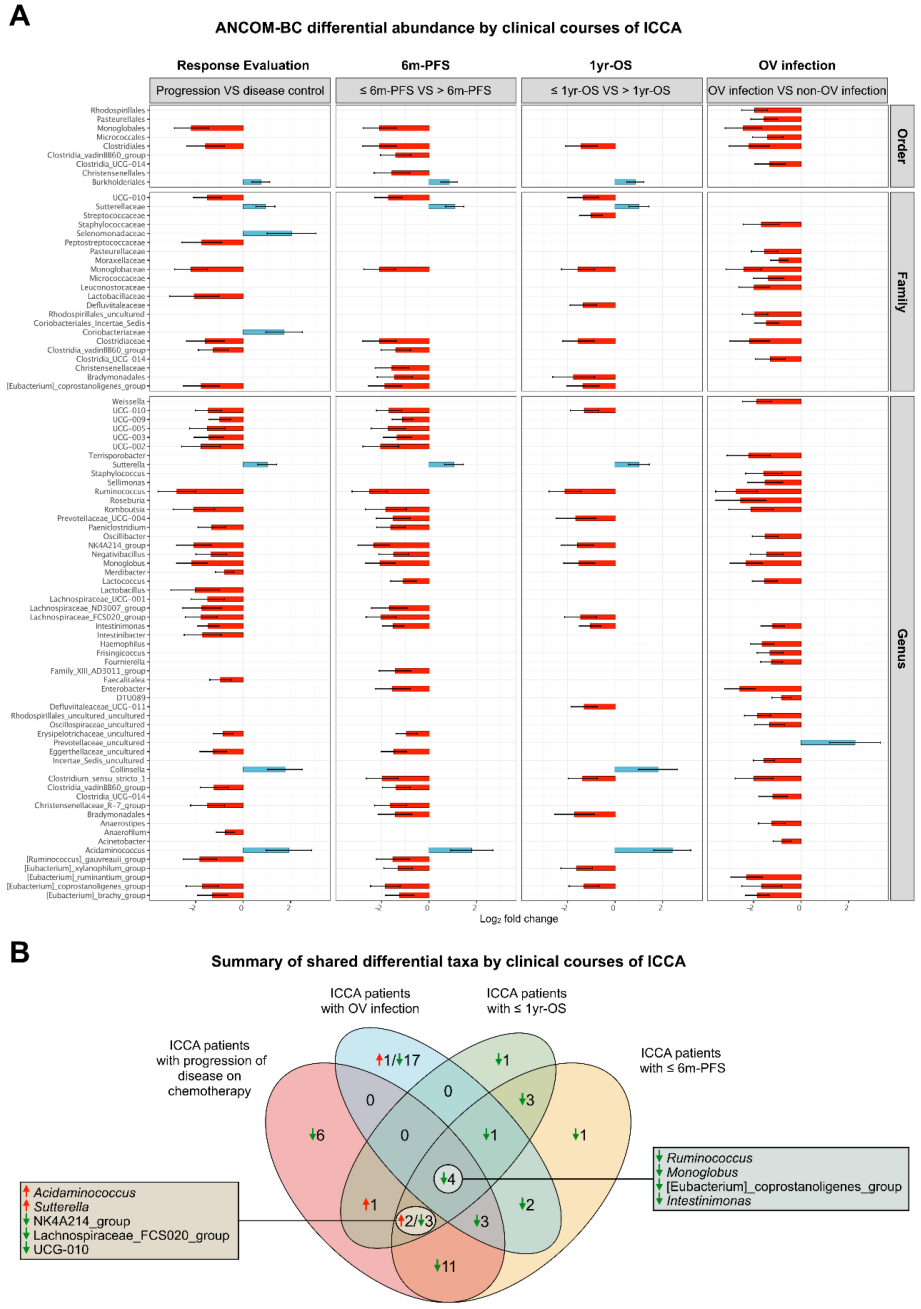
** (A)** Results of ANCOM-BC specifying the differential taxa between the groups categorized by response evaluation, 6m-PFS, 1yr-OS, and OV infection. A boxplot represents median with IQR, and each dot represents an outlier. **(B)** Venn diagram of the differential abundance of taxa. **Abbreviation:** ICCA, intrahepatic cholangiocarcinoma; OS, overall survival; OV, Opisthorchis viverrini; PFS, progression-free survival.

A taxonomic bar chart illustrates the relative abundance of bacterial taxa in the DCR and PD groups, highlighting differences in microbial composition (**Figures 2C**). When we compared gut microbiota profiles before receiving chemotherapy between the DCR group from CT evaluation and the PD group, the alpha-diversity index, as measured by Pielou's evenness index, was significantly decreased in the PD group compared to the DCR group. Beta-diversity indices, as measured by Unweighted UniFrac distance showed a significant difference in gut bacterial composition between the two groups (**Figure 2D**). ANCOM-BC analysis showed the differential abundance of bacterial taxa between two groups (**Figures 3A and Supplementary Table 4**).

### Gut microbiota could potentially differentiate prognosis in ICCA patients

A taxonomic bar chart illustrated the relative abundance of bacterial taxa in the DCR and PD groups, highlighting differences in microbial composition (**Figure 2C**). The Alpha-diversity indices, as indicated by Pielou's evenness, showed a significantly decreased gut bacterial populations in ≤ 6m-PFS group compared to > 6m-PFS group. Beta-diversity indices detected by Bray-Curtis dissimilarity showed a notable difference in gut bacterial composition between the two groups (**Figure 2E**). In contrast, Alpha-diversity and Beta-diversity indices showed no difference between ≤ 1yr-OS group and > 1yr-OS group (**Figure 2F**). However, ANCOM-BC analysis revealed the differential abundance of bacterial taxa between two groups (**Figure 3A and Supplementary Table 4**). Evaluating serum biochemical profiles demonstrated that absolute eosinophil count and creatinine levels were higher whereas the albumin level was lower in ≤ 6m-PFS, compared to > 6m-PFS group. Moreover, the absolute lymphocyte count was higher in > 1yr-OS while N/L ratio and P/L ratio were lower in > 1yr-OS group. There were no differences in oxidative stress, inflammatory markers (including IL-10, IL-1B, IL-6, MCP-1, and TNF-alpha), bile acids, and SCFAs (**Table 2**).

### Gut bacterial profiles were different between OV-infected patients and non-OV-infected patients with ICCA

OV positive patients were identified in nine out of 30 patients, accounting for 30% of the study population. Alpha-diversity and beta-diversity indices show no significant difference between the two groups (**Figure 2G**). ANCOM-BC analysis shows the differential abundance of bacterial taxa between the two groups (**Figure 3A and Supplementary Table 4**). Moreover, a comparison of basic laboratory results showed no difference between groups, except for propionic acid and butyric acid levels, which were significantly lower in the OV group.

### Identification of bacterial profile associated with poor prognosis

We determined bacterial profiles which shared ANCOM-BC differential abundance, as shown in **Figure 3B**. There were four species associated with PD from CT evaluation, ≤ 6m-PFS, ≤ 1yr-OS, and OV infection which were decreased including *Ruminococcus*, *Monoglobus*, [Eubacterium]_coprostanoligenes group, and *Intestinimonas spp*. In addition, consideration for only clinical outcomes (progression of disease as the best chemotherapy response, ≤ 6m-PFS, ≤ 1yr-OS), five species were identified. In this case, increased *Acidaminococcus* and *Sutterella* with decreased NK4A214_group, Lachnospiraceae_FCS020_group, and UCG-010 were associated with poor clinical outcomes (**Figure 3B**).

### Gut microbiota and its correlation with clinical parameters and biochemical profiles

**Figure 4:**
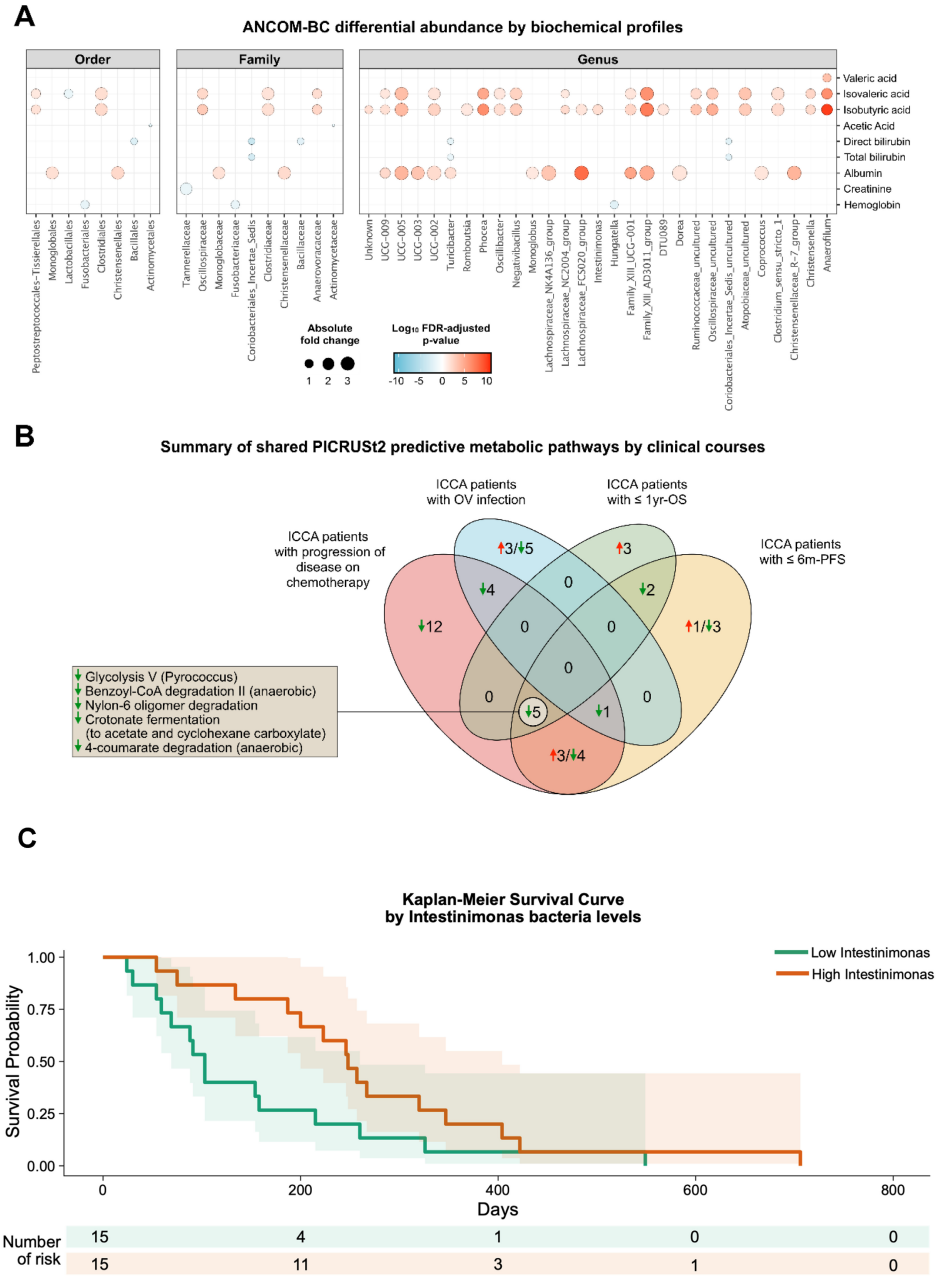
** (A)** ANCOM-BC indicated differential abundance throughout the taxonomic levels based on changes of each biochemical parameter. **(B)** Venn diagram of predictive metabolic pathway analysis. **(C)** Kaplan-Meier graph demonstrating PFS categorized by *Intestinimonas* levels. **Abbreviation:** ICCA, intrahepatic cholangiocarcinoma; OS, overall survival; OV, Opisthorchis viverrini; PFS, progression-free survival

ANCOM-BC showed the correlation of gut microbiota and clinical parameters including biochemical profiles (Figure 4A). Increased levels of serum isovaleric acid and isobutyric acid were positively associated with bacterial families Oscillospiraceae, Clostridiaceae, and Anaerovoracaceae, with genera such as *Intestinimonas*, *Ruminococcaceae*, and *Anaerofilum* (**Figure 4A and Supplementary Table 5**). Additionally, higher serum albumin levels were correlated with Monoglobaceae and Christensenellaceae families. At the genus level, Lachnospiraceae_FCS020_group and *Monoglobus* showed a strong correlation with elevated albumin. Other clinical parameters, including hemoglobin, creatinine, liver enzymes, and bilirubin did not show a significant correlation with gut microbiota profile.

### Shared predictive metabolite pathway associated with poor prognosis

Phylogenetic Investigation of Communities by Reconstruction of Unobserved States 2 (PICRUSt2) showed predicted alteration of metabolic pathways regarding microbiota change observed among patients with poor clinical outcomes. A decrease in glycolysis, benzoyl-CoA degradation, nylon degradation, croconate fermentation and 4-coumarate degradation were identified in poor clinical outcomes, including PD from CT evaluation, ≤ 6m-PFS, and ≤ 1yr-OS. (**Figure 4B and Supplementary Table 6**).

### Clinical and bacterial profile could help predict survival in ICCA patients

Univariate analysis using biochemical laboratory and gut microbiota profiles was performed to predict poor prognosis in ICCA patients following chemotherapy, including PD on CT evaluation, ≤ 6m-PFS, and ≤ 1yr-OS (**Supplementary Table 7**). Significant factors identified in the univariate analysis were further assessed in the multivariate analysis model (**Table 3**). UCG-010 and *Monoglobus* were significantly associated with PD on CT scan, while increased *Intestinimonas* level was significantly associated with > 6m-PFS with adjusted OR 0.92 (95% CI 0.84-0.99). In contrast, biochemical profile alone could not predict PD on CT evaluation and ≤ 6m-PFS. However, multivariate analysis incorporating both biochemical laboratory and gut microbiota profiles could not predict OS. Further exploration revealed *Intestinimonas* as a strong predictor of PFS with an AUC of 0.79. Using the cut-off level of 8.5, patients with a low level of fecal *Intestinimonas* had a mPFS of 3.39 months while those with high level had 8.15 months, with HR 0.49 (95% CI 0.23-1.02; p 0.058) (**Figure 4C**).

## Discussion

In the present study, we observed a decrease in alpha-diversity among patients who progressed on chemotherapy and those with ≤ 6m-PFS. These findings suggest that gut microbiota alterations, including a reduction in beneficial bacteria, contribute to gut dysbiosis, which may promote inflammation and carcinogenesis, as reported in many studies 9, 10. Notably, ICCA patients exhibited depletion of beneficial strain, *Saccharomyces cerevisiae*, and an overgrowth of pathologic fungi compared to healthy controls 10. Additionally, decreased abundances of *Ruminococcus*, *Monoglobus*, *Eubacterium*, and *Intestinimonas spp* were associated with PD on CT evaluation, ≤ 6m-PFS, ≤ 1yr-OS, and OV infection. Consistent with the findings of Ma et al. (2023), healthy controls had higher amount of *Ruminococcus, Porphyromonadaceae* and *Bacteroidota* compared to CCA and HCC patients, indicating that these taxa may serve as protective factors against cancer development 11. This hypothesis is supported by evidence showing that *Ruminococcus* and *Monoglobus* are well-known pectin-degrading bacteria 12, 13. Pectin, a dietary fiber, has been widely recognized for human health benefits, particularly mitigating metabolic diseases 12. Our study also identified correlations between gut microbiota and clinical parameters. Specifically, isovaleric acid and isobutyric acid were linked to *Ruminococcaceae* and *Intestinimonas* genera, suggesting that a decline in these bacteria, leading to reduced production of these SCFAs. A previous study reported that isobutyric acid exerted direct anti-tumor effect and enhanced T-cell mediated immunological process against cancer cells among patients receiving checkpoint inhibitors 14. Additionally, beneficial bacteria identified in stool samples, such as Lachnospiraceae_FCS020_group and Monoglobus, were positively associated with serum albumin levels. Pretreatment serum albumin levels have been widely recognized as a predictive biomarker for survival in cancer patients 15. Beyond its role as a nutritional marker, albumin also contributes to suppressing oxidative stress, further highlighting its multifaceted significance in cancer treatment 15, 16. Moreover, an increase in *Acidaminococcus* and *Sutterella* together with a decrease in NK4A214_group, Lachnospiraceae _FCS020_group, and UCG-010 were associated with poor clinical outcomes (PD on CT evaluation, ≤ 6m-PFS, ≤ 1yr-OS). NK4A214_group and UCG-010*,* both members of the *Ruminococcaceae* family, are known SCFAs producers, particularly butyrate 17. Butyrate is crucial in maintaining gut barrier integrity, reducing inflammation, and modulating intestinal motility 18, 19. A reduction in these bacteria can result in reduced butyrate production, potentially compromising gut barrier function. This, in turn, could facilitate gut leakage, allowing lipopolysaccharide (LPS)-containing bacteria and gut metabolites to trigger inflammation, ultimately contributing to the progression of CCA 4. Notably, similar reductions in these bacteria have been observed in colorectal cancer, where their levels were significantly lower compared to healthy controls 20. Nevertheless, the relatively small sample size may limit the ability to draw definitive conclusions regarding the biological relevance of the identified microbial taxa.

Our study demonstrated that OV-infected ICCA patients exhibited lower levels of fecal propionic and butyric acid compared to non-OV-infected patients. This suggests a depletion of butyrate-producing bacteria in OV-infected patients, potentially contributing to disease progression. Additionally, *Lachnospiraceae*, a beneficial bacterial genus commonly found in individuals following a healthy diet, was also linked to poor survival outcomes. Its reduction has correlated with adverse metabolic profiles and elevated inflammatory biomarkers, potentially exacerbating the inflammatory environment and contributing to disease progression 12. Together, gut microbiota alterations, particularly the reduction of beneficial bacteria, could lead to poor survival outcomes in advanced ICCA patients receiving chemotherapy.

Metabolic pathway alterations associated with poor clinical outcomes included reductions in glycolysis, benzoyl-CoA degradation, nylon-6-oligomer degradation, crotonate fermentation, and 4-coumarate degradation. In general, glycolysis in cancer cells supports cancer proliferation and chemotherapy resistance, and several studies have identified it as a potential target to enhance treatment efficacy 21. While reduction of host metabolic glycolysis is distinct, this can impair cancer cell energy production and alter the tumor microenvironment, potentially influencing cancer progression 22. Among these metabolic pathways, benzoyl-CoA degradation is particularly relevant due to its association with inflammatory processes and insulin resistance. In patients with type 2 diabetes, benzoyl-CoA has been shown to inhibit insulin secretion, trigger inflammatory cytokines, and induce β-cell apoptosis 23. Therefore, a reduction in benzoyl-CoA degradation may exacerbate inflammatory responses, contributing to cancer progression and poorer survival outcomes. The relationship between these metabolic shifts underscores the complex interplay between metabolic regulation, inflammation and oncogenesis.

Multivariate analysis highlights gut microbiota as predictive biomarkers of chemotherapy. Reduced UCG-010 and *Monoglobus* levels were associated with disease progression on CT scans, while decreased *Intestinimonas* levels correlated with ICCA progression within six months. Notably, an* Intestinimonas* level below 8.5 was associated with poorer prognosis in advanced ICCA in our study; however, this finding should be interpreted cautiously given the borderline statistical significance and lack of validation. *Intestinimonas* has been recognized as protective against non-alcoholic fatty liver disease 24 and was shown to increase following temozolamide chemotherapy in a glioma mouse model. This correlates with sesquiterpenoid and triterpenoid biosynthesis, resulting in anti-tumor and antioxidant activities 25. Given the increasing implementation of immune checkpoint inhibitors in solid malignancies, including recently approved therapy in CCA, the role of gut microbiota in modulating treatment response is of particular interest. In biliary tract cancer patients receiving anti-PD1/PD-L1 therapy, higher *Alistipes* levels were associated with significantly improved PFS and OS. A decline in *Alistipes* may reduce SCFA production, diminishing anti-inflammatory effects and reducing checkpoint inhibitor efficacy 26. Our findings suggest that gut *Intestinimonas* levels may serve as a prognostic biomarker for distinguishing survival outcomes in advanced ICCA patients.

However, this study did not detect significant differences in the quantification of selected bacterial taxa in cancerous tissues between ICCA and HCC patients. Even after adjusting for major clinical factors, including cirrhosis status, HBV infection, HCV infection, and disease stage (metastatic vs non-metastatic), the results remained unchanged, with no significant differences observed in tissue bacterial abundance between the two groups. These findings should be interpreted with caution, as bacterial assessment in tumor tissues was performed using a targeted RT-qPCR panel focusing on selected taxa rather than an untargeted microbiome profiling approach, and tumor tissues typically contain low bacterial biomass. In contrast, gut microbiota analysis in advanced ICCA patients prior to chemotherapy revealed that patients with poor clinical outcomes exhibited decreased microbial diversity and distinct gut microbial compositions. Additionally, ANCOM-BC and PICRUSt2 analyses supported differential bacterial taxa and predicted metabolic pathways between the good- and poor-outcome groups.

Currently, limited information exists regarding the intertumoral microbiota differences between ICCA and HCC. Distinct contributing factors, such as liver fluke infection, can alter microbiota along the gut-bile axis, potentially leading to microbiota changes within tumors and reflecting inflammatory processes in carcinogenesis. Previous studies reported an increased presence of *Helicobacter* spp. in biliary tract cancer tissues compared to benign biliary diseases 27-29. While migration of gut microbiota to the biliary system may plausibly affect extrahepatic region, it remains unclear whether these microorganisms colonize in intrahepatic tumors. To date, no study has directly compared microbiota profiles between CCA and HCC.

In the first cohort, we found no differences in representative gut bacterial taxa in cancerous tissues between ICCA and HCC. A previous study examining microbiota profiles in CCA tumor tissues reported increased levels of *Bifidobacteriaceae* and *Enterobacteriaceae* in OV-infected tumors but no significant microbiota differences between tumor and adjacent liver tissue in OV-infected ICCA. However, in non-OV-infected ICCA, increased levels of *Stenotrophomonas* and *Xanthomonadaceae* were identified 5. These findings suggest that further investigation at the species level may yield more insightful conclusions, as our study could only evaluate the tumor tissue at the phylum level. Although bacterial composition in cancerous liver tissues remains poorly understood, gut microbiota is of particular interest. Recent studies in Thai patients have reported no differences in the gut microbiota profiles at the phylum and family levels between ICCA and HCC; however, the differences were detected at the species level. Furthermore, alpha-and beta-diversity measures did not reveal significant differences between ICCA, HCC, and healthy individuals 30. In contrast, a study in a Chinese population reported differences at the phylum level in fecal samples with HCC patients exhibiting lower *Bacteroidota* and higher *Actinomicrobiota* abundance compared to ICCA patients and healthy controls 31. These findings suggest that gut microbiota may hold potential as a biomarker for distinguishing between ICCA and HCC diagnosis.

### Limitations of Study

Future research should incorporate a larger patient cohort and employ shotgun sequencing for characterization of the intertumoral microbiota in both ICCA and HCC. In addition, the inherently low bacterial DNA yield from cancerous tissues, even when fresh specimens are obtained, presents a challenge in sample collection and processing for tissue microbiome studies. Beyond chemotherapy, the recent approval of combination therapies integrating chemotherapy and immune checkpoint inhibitors underscores the need to investigate the gut and cancerous microbiota profiles associated with clinical outcomes following these regimens. A deeper understanding of these microbiota alterations could improve patients' outcomes. Furthermore, elucidating the mechanistic pathways through which microbiota influence treatment response and patients' prognosis in primary liver cancers, including both CCA and HCC, remains a critical area for further investigations. In addition, the relatively small sample size in the chemotherapy cohort limited the number of predictors that could be included in multivariable models relative to the number of outcome events, and therefore the models were intentionally kept parsimonious to reduce the risk of overfitting. Moreover, internal validation procedures such as bootstrap resampling or cross-validation were not performed due to the limited sample size; therefore, the findings should be interpreted as exploratory and require validation in larger independent cohorts.

## Conclusion

Although bacterial profiles of cancerous tissues did not differ between ICCA and HCC, advanced ICCA patients with different prognoses during follow-ups exhibited distinct gut microbiota profiles at baseline prior to receiving chemotherapy. These findings highlight the potential roles of gut microbiota as prognostic markers in advanced ICCA for predicting clinical outcomes.

## Supplementary Material

Supplementary materials and methods and tables.

## Figures and Tables

**Table 1:** Baseline Demographic and Clinical Characteristics of ICCA Patients Before Receiving Chemotherapy.

Characteristics	ICCA patients (n=30)								
	6m-PFS		p-value	1yr-OS		p-value	OV infection		p-value
	> 6m-PFS (n= 16)	≤ 6m-PFS (n=14)		> 1yr-OS (n=12)	≤ 1yr-OS (n=18)		Non-OV (n=21)	OV (n=9)	
**Demographics**									
Age, year	63.9 (56.7 -71.1)	61.1 (51.9 - 70.3)	0.36	62.9 (55.0-70.9)	62.3 (53.8-70.9)	0.85	61.2 (52.5-69.9)	65.7 (59.6-71.8)	0.16
Sex, n (%)			0.08			0.15			0.44
Male	6 (37.5)	10 (71.4)		4 (33.3)	12 (66.7)		10 (47.6)	6 (66.7)	
Female	10 (62.5)	4 (28.6)		8 (66.7)	6 (33.3)		11 (52.4)	3 (33.3)	
BW, kg (mean, SD)	56.1 (46.6-65.6)	56.1 (44.6-67.6)	1.00	57.6 (48.0-67.2)	55.1 (44.3-65.9)	0.52	56.6 (47.5-65.7)	54.8 (41.7-67.9)	0.65
Risk factor									
Smoking (%)	5 (31.3)	2 (14.3)	0.40	4 (33.3)	3 (16.7)	0.54	5 (23.8)	2 (22.2)	1.00
Alcohol consumption (%)	4 (25.0)	4 (28.6)	1.00	3 (25.0)	5 (27.8)	1.00	5 (23.8)	3 (33.3)	0.67
Raw food consumption (%)	2 (12.5)	4 (28.6)	0.38	2 (16.7)	4 (22.2)	1.00	5 (23.8)	1 (11.1)	0.64
Cirrhosis (%)	1 (6.3)	2 (14.3)	0.58	1 (8.3)	2 (11.1)	1.00	0	3 (33.3)	0.21
HBV infection (%)	1 (6.3)	1 (7.1)	1.00	1 (8.3)	1 (5.6)	1.00	1 (4.8)	1 (11.1)	0.52
HCV infection (%)	1 (6.3)	0	1.00	1 (8.3)	0	0.4	1 (4.8)	0	1.00
Antibiotics use (%)	0	1 (7.1)	0.47	0	1 (5.6)	1.0	0	1 (11.1)	0.30
OV (%)	2 (12.5)	7 (50.0)	**0.045**	2 (16.7)	7 (38.9)	0.37	0	100	
**Tumor staging**									
T (%)			0.06			0.11			0.14
1-2	11 (78.8)	7(50.0)		8 (66.7)	10 (55.5)		12 (57.1)	6 (66.7)	
3-4	5 (31.2)	7(50.0)		4 (33.3)	8 (44.5)		9 (42.9)	3 (33.3)	
N (%)			0.90			1.0			1.00
0	1 (6.3)	2 (14.3)		1 (8.3)	2 (11.1)		2 (9.5)	1 (11.1)	
1	15 (93.8)	12 (85.7)		11 (91.7)	16 (88.9)		19 (90.5)	8 (88.9)	
M (%)			0.60			1.0			1.00
0	3 (18.7)	1 (7.1)		2 (16.7)	2 (11.1)		3 (14.3)	1 (11.1)	
1	13 (81.3)	13 (92.9)		10 (83.3)	16 (88.9)		18 (85.7)	8 (88.9)	
Metastatic site (%)			0.44			0.69			0.12
0-1	12 (75.0)	8 (57.1)		9 (75.0)	11 (61.1)		16 (76.2)	4 (44.4)	
≥ 2	4 (25.0)	6 (42.9)		3 (25.0)	7 (38.9)		5 (23.8)	5 (55.6)	
ORR (%)			**<0.01**			**<0.01**			0.14
Partial response	3 (18.8)	1 (7.1)		1 (8.3)	3 (16.7)		3 (14.3)	1 (11.1)	
Stable of disease	13 (81.3)	1 (7.1)		11 (91.7)	3 (16.7)		12 (57.1)	2 (22.2)	
Progression of disease	0	12 (85.7)		0	12 (66.7)		6 (28.6)	6 (66.7)	
**Tumor markers**									
CEA (U/ml)	6.5 (3.2,43.5)	6.0 (2.8,10.7)	0.86	6.5 (3.2,43.5)	6.0 (3.1,10.0)	0.44	6.4 (3.2,43.5)	10.7 (2.8,11.7)	0.85
CA 19-9 (U/ml)	455.2 (16.3,3170)	67.8 (9.7,2540)	0.84	576.0 (17.4,2986.0)	490.3 (21.4,2492.8)	0.62	736.7 (56.4,2976)	103.5 (14.0,856.2)	0.29

**Abbreviations**: BW, body weight; CA 19-9, cancer antigen 19-9; CEA, carcinoembryonic antigen; HBV, hepatitis B virus; HCV, hepatitis C virus; ICCA, intrahepatic cholangiocarcinoma; kg, kilogram; ORR, objective response rate; PFS, progression-free survival; OS, overall survival; OV, *Opisthorchis viverrini.*

**Table 2:** Blood Biochemical Profiles of Advanced ICCA Patients Receiving Chemotherapy

Biochemical parameters	> 6m-PFS (n = 16)	≤ 6m-PFS (n = 14)	p-value	> 1yr-OS (n = 12)	≤ 1yr-OS (n = 18)	p-value	Non-OV (n = 21)	OV (n = 9)	p-value
**Basic laboratory parameters**									
Hb (g/dl, IQR)	12.1 (11.2,12.7)	12.3 (11.8,13.0)	0.72	12.1 (10.6,13.7)	12.0 (11.1,13.0)	0.72	12.0 (11.4,12.5)	12.7 (12.2,13.5)	0.18
WBC (n,SD)	8839.4 (5856.3 - 11822.4)	9021.4 (6009.6-12033.2)	0.87	9035.8 (6254.1- 11817.6)	8850.0 (5722.5- 11977.5)	0.87	9523.8 (6471.6 - 12576.1)	7525.6 (5284.8-9766.3)	0.09
ANC (n, IQR)	5504.7 (4586.8,6585.0)	6414.0 (4460.7,7354.8)	0.74	5784.5 (3802.8,7766.1)	6021.3 (3690.0,8352.6)	0.77	6311.8 (5068.3,7910.8)	4854.9 (3550.0,5904.2)	0.08
ALC (n, IQR)	2012.9(1552.0,2643.3)	1701.4 (1178.9,2054.2)	0.10	2194.4 (1851.0,2849.4)	1678.6 (1178.9,1931.9)	**0.02**	1889.5 (1413.6,2528.1)	1759.6 (1350.2,2208.8)	0.86
AEC (n, IQR)	195.4 (152.7,365.6)	642.8 (156,988.4)	**0.04**	172.6 (152.7,342.6)	282.0(156.1,582.7)	0.10	248.4 (144.0,426.7)	230.7 (157.0,496.3)	0.86
Platelet (n, IQR)	288000 (261250, 365000)	325500 (265500, 402500)	0.45	274500.0 (261250.0, 321500.0)	328000.0 (265500.0, 379750.0)	0.34	320000 (267100, 380000)	273000 (216000, 216000)	0.10
N/L ratio (IQR)	2.7 (1.9,3.3)	3.5 (2.5,3.9)	0.19	2.2 (1.8,2.9)	3.5 (2.8,3.9)	**0.02**	3.2 (2.2,4.0)	2.8 (2.1,3.5)	0.40
P/L ratio (IQR)	144.4 (102.9,200.9)	186.8 (156.1,232.9)	0.44	116.9 (97.6,182.6)	199.2 (159.0,232.9)	**0.02**	177.4 (123.3-235.4)	158.3 (112.2-198.3)	0.50
Creatinine (mg/dL, IQR)	0.8 (0.7,0.9)	1.0 (0.7,1,2)	**0.04**	0.8 (0.7,0.9)	0.9 (0.7,1,2)	0.36	0.8 (0.7,1.0)	0.9 (0.7,1.2)	0.51
Alb (g/dL, IQR)	4.0 (3.9,4.2)	3.7 (3.5,4.0)	**0.03**	4.0 (3.8,4.2)	3.9 (3.5,4.0)	0.42	3.9 (0.4)	3.8 (0.4)	0.41
ALP (U/L, IQR)	182.0 (131.3,446.0)	267.0 (248.3,405.3)	0.22	182.0 (142.5,432.5)	267.0 (229.3,433.8)	0.30	288.0 (173,436)	258.0 (205,270)	0.62
AST (U/L, IQR)	46.0 (24.5,55)	43.5 (36.3,64.8)	0.52	46.0 (28.8,52.3)	43.5 (30.0,65.0)	0.54	44.0 (25,61)	48.0 (36,64)	0.62
ALT (U/L, IQR)	38.0 (17.5,66.8)	47.0 (23,64.8)	0.51	38.0 (19.0,57.8)	47.0 (22.3,77.0)	0.54	49.0 (22,66)	23.0 (20,64)	0.77
TB (mg/dl, IQR)	0.7 (0.6,1.5)	0.8 (0.6,1.4)	0.84	0.7 (0.6,1.6)	0.7 (0.5,1.3)	1.00	0.7 (0.5,1.5)	0.8 (0.7,0.9)	0.56
DB (mg/dl, IQR)	0.3 (0.2,0.8)	0.4 (0.3,0.9)	0.72	0.4 (0.3,1.4)	0.4 (0.3,0.7)	0.88	0.4 (0.3,1.0)	0.4 (0.3,0.7)	0.87
**Inflammatory markers**									
Oxidative stress (IQR)	20194.0 (17471, 25919)	19427.0 (16574,30821)	0.72	20543.0 (17561.5,25531.8)	18852.5 (16424.3, 31018.5)	0.82	20194.0 (17532.5,30709.0)	17124.0 (15823.5,24210.5)	0.36
IL-10 (IQR)	2.7 (2.2,7.4)	3.1 (1.8,5.0)	0.69	2.7 (2.3,7.4)	3.1 (1.8,5.0)	0.56	3.3 (2.3,7.0)	2.2 (1.8,3.1)	0.19
IL-1B (IQR)	0.9 (0.7,4.8)	0.9 (0.6,1.0)	0.30	0.9 (0.8,3.9)	0.8 (0.6,1.2)	0.15	0.9 (0.7,2.1)	0.9 (0.6,1.0)	0.59
IL-6 (IQR)	4.3 (1.9,8.8)	3.5 (0.9,5.9)	0.33	4.3 (1.9,7.3)	3.5 (1.1,6.4)	0.56	4.3 (1.9,6.6)	2.3 (0.9,4.4)	0.40
MCP-1 (IQR)	292.6 (165.4,387.8)	1216.9 (179.1,445.8)	0.85	292.6 (165.4,387.8)	216.9 (179.1,445.8)	0.78	292.6 (177.9,437.9)	216.9 (171.2,351.5)	0.53
TNF-alpha (IQR)	15.0 (10.3,23.1)	11.5 (8.1,22.7)	0.35	15.0 (11.6,23.0)	11.5 (8.1,22.7)	0.32	13.4 (9.8,21.7)	12.0 (8.2,29.2)	0.98
**Bile acids**									
Chenodeoxycholic acid (IQR)	998.8 (26.5,2148.6)	762.9 (72.7,1506.9)	0.79	358.3 (0.0,2168.5)	1087.9 (247.5,1507.6)	0.69	1349.1 (152.5,2139.1)	185.2 (0,1199.8)	0.13
Cholic acid (IQR)	66.7 (9.8,163.0)	65.67 (18.4,230.6)	0.66	38.7 (0.0,152.5)	82.8 (27.2,177.2)	0.30	87.3 (37.4,247.1)	23.3 (0.0,62.3)	0.08
Glycocholic acid (IQR)	971.8 (645.8,2445.4)	2240.5 (1739.8,3441.5)	0.09	1214.0 (760.8,2445.4)	2208.5 (820.4,3441.5)	0.39	1299.1 (647.5,2839.4)	2261.6 (1749.0,3214.3)	0.30
Glycodeoxycholic acid and glycoursodeoxycholic acid (IQR)	1138.4 (342.8,2033.0)	2079.7 (1038.9,3034.3)	0.18	1066.6 (321.9,2033.0)	1640.8 (985.1,3034.3)	0.22	1274.8 (786.8,2488.1)	1935.3 (550.0,3028.9)	1.00
Taurochenodeoxycholic acid and tauroursodeoxycholic acid (IQR)	191.7 (76.1,448.3)	359.0 (277.9,1239.3)	0.11	116.3 (73.6,394.8)	359.0 (253.3, 997.9)	0.07	364.1 (117.2,788.8)	267.2 (115.5,353.9)	0.62
Taurocholic acid (IQR)	250.2 (83.2,649.6)	500.0 (256.8,1235.3)	0.08	237.1 (66.8,649.6)	423.0 (234.9, 1096.8)	0.13	307.6 (180.9,997.5)	464.9 (194.0,1114.3)	0.69
**Short-chain fatty acids**									
Acetic acid	5.1 (3.3,6.9)	5.3 (2.5,7.1)	0.85	5.1 (3.0,7.5)	5.3 (2.6,6.9)	0.88	5.4 (4.5,8.1)	2.3 (1.7,7.0)	0.09
Propionic acid	2.6 (2.1,3.5)	2.4 (1.5,3.9)	0.81	2.8 (2.1,3.5)	2.5 (1.5,3.5)	0.74	2.7 (2.3,4.0)	1.4 (1.0,2.6)	**0.04**
Isobutyric acid	0.3 (0.2,0.6)	0.3 (0.2,0.5)	0.98	0.4 (0.2,0.6)	0.3 (0.2,0.5)	0.84	0.3 (0.2,0.7)	0.3 (0.2,0.4)	0.36
Butyric acid	1.9 (1.0,3.0)	1.5 (0.8,2.9)	0.98	1.9 (0.8,2.4)	1.5 (1.0,3.2)	0.77	2.1 (1.1,3.7)	0.7 (0.3,1.7)	**0.04**
Isovaleric acid	0.3 (0.2,0.6)	0.4 (0.2,0.5)	0.98	0.4 (0.2,0.6)	0.3 (0.2,0.5)	0.84	0.3 (0.2,0.7)	0.3 (0.2,0.4)	0.63
Valeric acid	0.4 (0.1,1.0)	0.4 (0.1,0.7)	0.56	0.2 (0.1,1.0)	0.4 (0.1.07)	0.88	0.6 (0.1,0.9)	0.1 (0.0,0.6)	0.18

**Abbreviations**: Alb, albumin; ALP, alkaline phosphatase; AST, *aspartate aminotransferase*; ALT, alanine transaminase; AEC, absolute eosinophil count; ALC, absolute lymphocyte count; ANC, absolute neutrophil count; DB, direct bilirubin; Hb, hemoglobin; ICCA, intrahepatic cholangiocarcinoma; IL, interleukin; IQR, interquartile range; MCP-1, *monocyte chemoattractant protein*-*1*, N/L, neutrophil per lymphocyte; ORR, objective response rate; OS, overall survival; OV, Opisthorchis viverrini; PFS, progression-free survival; P/L, platelet per lymphocyte; TB, total bilirubin; TNF, tumor necrotic factor; WBC, white blood count.

**Table 3:** Multivariate Analysis of Biochemical Laboratory and Bacterial Profiles Identifying Poor Prognosis of ICCA Patients Receiving Chemotherapy

Characteristics	Response evaluation				6m-PFS				1yr-OS			
	Individual effect		Interaction effect		Individual effect		Interaction effect		Individual effect		Interaction effect	
	aOR (95% CI)	p-value	aOR (95% CI)	p-value	aOR (95% CI)	p-value	aOR (95% CI)	p-value	aOR (95% CI)	p-value	aOR (95% CI)	p-value
**Biochemical profiles**												
Absolute lymphocyte count	NA	NA	1.4 (0.9-1.0)	> 0.05	NA	NA	NA	NA	1.0 (0.9-1.0)	> 0.05	0.2 (0.0-0.5)	> 0.05
Lymphocyte	NA	NA			NA	NA			1.1 (0.9-1.3)	> 0.05		
Eosinophils	1.4 (1.0-2.3)	>0.05			NA	NA			NA	NA		
Platelet-to-lymphocyte ratio	NA	NA			NA	NA			1.0 (0.9-1.0)	> 0.05		
Creatinine	7.5 (0.3-295.3)	> 0.05			NA	NA			NA	NA		
**Gut microbiota**												
NK4A214_group	1.0 (0.9-1.0)	> 0.05	1.00 (0.99-1.01)	> 0.05	NA	NA	0.9 (0.9-1.0)	> 0.05	NA	NA	NA	NA
Lachnospiraceae_FCS020_group	NA	NA			0.9 (0.9-1.0)	> 0.05			NA	NA		
UCG-010	1.0 (1.0-1.0)	**< 0.05**			0.9 (0.9-1.0)	> 0.05			NA	NA		
*Monoglobus*	1.1 (1.0-1.0)	**< 0.05**			1.0 (0.9-1.0)	> 0.05			NA	NA		
*20_coprostanoligenes_group*	NA	NA			1.0 (0.9-1.0)	> 0.05			NA	NA		
*Intestinimonas*	NA	NA			0.9 (0.8-1.0)	**< 0.05**			NA	NA		

**Abbreviations**: NA, not applicable; OS, overall survival; OV, Opisthorchis viverrini; PFS, progression-free survival.

## Data Availability

Sequencing data are publicly available in the NCBI Sequence Read Archive (SRA) under BioProject accession number PRJNA1434637 (sample accessions SRR37547124-SRR37547153).
