# Supplementary material for: Prognostic Role of Gut Microbiota and Clinical Parameters in Predicting Survival in Advanced Cholangiocarcinoma Patients Receiving Chemotherapy
Source: Int J Med Sci. 2026 Jun 10;23(7):2448–61. doi: 10.7150/ijms.131376 (PMC13280742; doi:10.7150/ijms.131376)
Supplement: Supplementary file 1 — Supplementary materials and methods and tables. [file ijmsv23p2448s1.pdf]

Supplementary document

Laboratory Measurement

Collection and processing of biospecimens

For Cohort one, the 1 cm x 0.5 cm fresh cancerous liver tissue was be collected at the time of core-needle biopsy or surgery. For Cohort two, before patients received systemic chemotherapy, participants had been directed to collect their feces using a fecal collection kit for gut microbiome study and targeted metabolomics of fecal short-chain fatty acids (SCFAs). Laboratory process and testing was described in supplementary document. Ten milliliters of whole blood were collected to investigate basic biochemical profile, oxidative stress, inflammatory markers, bile acids, and serum ferritin. Quantification of serum ferritin and the basic biochemical profile were conducted using the Cobas e801 immunoassay analyzer (Roche Diagnosis GmbH, Mannheim, Germany). Plasma was prepared by centrifuging whole blood at 3000 rpm for ten minutes. All biospecimens were then preserved at -80°C.

Gut microbiota

Bacterial genomic DNA was extracted from 0.25 g of fecal samples using the QIAamp PowerFecal Pro DNA Kit (Qiagen, Hilden, Germany) according to the manufacturer's instructions. The V3–V4 hypervariable regions of the 16S rRNA gene were amplified using the 341F and 805R primers and sequenced using paired-end reads on the Illumina NovaSeq 6000 platform (Novogene, Singapore) (1). Raw sequencing reads were processed using Quantitative Insights into Microbial Ecology 2 (QIIME 2, v.2023.5). Sequence denoising, quality filtering, chimera removal, and amplicon sequence variant (ASV) generation were performed using the DADA2 plugin (2-4). Taxonomic classification of ASVs was conducted using a pretrained

classifier against the SILVA database (version 138) (5). Samples were rarefied to a minimum sequencing depth of 77,443 reads per sample to retain all samples for downstream diversity analyses. Alpha- and beta-diversity analyses were subsequently performed using QIIME2. To reduce potential noise from extremely rare taxa, taxa present in less than 10% of samples were excluded prior to differential abundance analysis. For differential abundance analysis, QIIME2 outputs were imported into phyloseq environment in R. Taxonomic features were aggregated at multiple taxonomic levels (phylum, class, order, family, and genus). Differentially abundant taxa between groups were identified using Analysis of Compositions of Microbiomes with Bias Correction (ANCOM-BC) (6). The statistical model included the group variable as the main effect. Multiple-testing correction was applied using the Bonferreni method, and taxa with adjusted p-values < 0.05 were considered statistically significant. Visualization of differential abundance results was performed using custom R scripts based on log fold changes estimated by ANCOM-BC.

Functional pathway prediction was performed using PICRUSt2 with the MetaCyc database as the reference pathway database. Predicted MetaCyc pathway abundance tables were analyzed in R using ggpicrust2. To reduce noise from sparse features, pathways with nonzero abundance in fewer than three samples were removed prior to differential abundance analysis. Differentially abundant pathways between groups were identified using Linear Models for Differential Abundance (LinDA), with the group variable specified as the main effect and a reference group. Pathway annotations were assigned using the MetaCyc database, and significant pathways were exported for Venn diagram analysis (7, 8). Associations between gut microbial taxa and continuous clinical or metabolite variables were evaluated using ANCOM-BC in R. QIIME2-derived feature tables were imported into the phyloseq environment and aggregated at the

phylum, class, order, family, and genus levels. For each clinical or metabolite variable, an independent ANCOM-BC model was fitted with the variable treated as a continuous predictor. ANCOM-BC estimates taxon-specific log-fold changes using a bias-corrected generalized linear model framework that accounts for the compositional nature of microbiome data. Multiple hypothesis testing was controlled using Bonferroni correction, and taxa with adjusted p-values < 0.05 were considered statistically significant. Estimated log-fold changes and corresponding standard errors were extracted for visualization and interpretation.

Tissue bacterial quantification

Genomic DNA was extracted from cancerous liver tissue using the DNeasy Blood and Tissue Kit (Qiagen, Hilden, Germany) according to the manufacturer's instructions. Extracted DNA was normalized to a concentration of 50 ng/uL prior to downstream analyses. Representative bacterial taxa were quantified using quantitative real-time PCR (RT-qPCR), including Eubacteria (total bacterial load), Firmicutes (estimated from the combined abundance of Clostridiales and *Lactobacillus acidophilus*), Bacteroidota, and Enterobacteriaceae, following previously established protocols. RT-qPCR assays were performed in accordance with the Minimum Information for Publication of Quantitative Real-Time PCR Experiments (MIQE) guidelines (9). Standard curves were generated using serial dilutions of plasmids containing the target gene sequences from reference bacterial strains, and the standards were included in the same reaction plate as the study samples. No-template controls (NTC) were included in each run to monitor potential contamination during PCR amplification. Differences in bacterial composition between intrahepatic cholangiocarcinoma (ICCA) and hepatocellular carcinoma (HCC) tissues were

evaluated based on the abundance of these taxa together with the Firmicutes/Bacteroidota (F/B) ratio (10).

Determination of Opisthorchis viverini infection

Serum sample in 50 µL was used to diagnose OV infection by immunochromatographic point-of-care testing kit (11).

Oxidative stress measurement in peripheral blood mononuclear cells

Three milliliters of blood were used for peripheral blood mononuclear cells (PBMCs) isolation. Reactive oxygen species is a marker of oxidative stress, to determine ROS production in PBMCs, PBMCs were incubated with 2 µM Dichlorohydro-Fluorescein Diacetate dye (DCFH-DA), at 25°C for 20 minutes. The DCFH-DA diffused through the mitochondrial cell membrane, and it was deacetylated by intracellular esterases to a non-fluorescent compound, which was later oxidized by ROS into a dichlorofluorescein (DCF) form. DCF was a highly fluorescent compound, which can be detected by fluorescence spectroscopy with maximum excitation. An increased level of DCF indicates an increased ROS production. Fluorescent intensity of the DCF was measured with an excitation wavelength at 485 nm, and an emission wavelength at 530 nm using a flow cytometer (FACS Celesta, BD biosciences, San Jose, CA, USA) (12, 13).

Inflammatory cytokine expression

The expression of tumor necrosis factor (TNF)-α, transforming growth factor (TGF)-β, interleukin (IL)-1β, and IL-6 genes were measured by SYBR Green-based Quantitative Reverse Transcription Polymerase Chain Reaction (RT-qPCR) of mRNA extracted from the buffy coated

blood. The thermal cycling conditions were used denaturation at 95°C for 20 s, alignment at 54°C for 20 s, and elongation at 72°C for 20 s, for 40 cycles. A second pair of beta-actin primers were used as an internal control: forward, 5’-CCAGATCATGTTTGAGACC-3’ and reverse, 5’-ATGTCACGCACGATTTCCC-3’. All reactions were performed in duplicate. Reaction mixtures, without RNA, were used as negative controls in each run (13).

Bile acid analysis

Bile acid concentrations were quantitated using a 1260 infinity II liquid chromatography/6546 quadrupole time-of-flight mass spectrometry (Agilent technologies, Santa Clara, CA, USA) under reversed phase liquid chromatography negative ion mode. Mobile phase A was 0.1% formic acid in water and mobile phase B was 0.1% formic acid in acetonitrile. The column setting and gradient of mobile phases were described in a previous study (14). The mass spectrometry setting was as follows: dual Agilent jet stream electrospray ionization, full-scan mass spectrometry detection (m/z 50 to 1,200), acquisition rate 1 spectrum/sec, capillary voltage 3,500 V, nozzle voltage 1,500 V, gas temperature 350 °C, drying gas 10 L/min, nebulizer pressure 20 psig, and reference mass correction was enabled (15).

Targeted metabolomics of SCFAs

Gas chromatography-mass spectrometry has been primarily designated for use in targeted metabolomics, specifically for SCFAs (16). To quantify the targeted metabolomics of SCFAs in both fecal content and plasma, gas chromatography-mass spectrometry (Agilent Technologies, California, USA) was employed, as described in our previous study (4). The actual concentration of SCFAs was calculated by comparison with a standard mixture (Restek, Pennsylvania, USA)

using MassHunter Quantitative Analysis Software v.10.1 (Agilent Technologies, California, USA) (14).

References

1. Sriwichaiin S, Kittichotirat W, Chunchai T, Chattipakorn N, Chattipakorn SC. Profiles of gut microbiota in obese-insulin-resistant rats treated with biotics. Eur J Nutr. 2022.

2. Weiss S, Xu ZZ, Peddada S, Amir A, Bittinger K, Gonzalez A, et al. Normalization and microbial differential abundance strategies depend upon data characteristics. Microbiome. 2017;5(1):1-18.

3. Bolyen E, Rideout JR, Dillon MR, Bokulich NA, Abnet CC, Al-Ghalith GA, et al. Reproducible, interactive, scalable and extensible microbiome data science using QIIME 2. Nat Biotechnol. 2019;37(8):852-7.

4. Sriwichaiin S, Thiennimitr P, Thonusin C, Sarichai P, Buddhasiri S, Kumfu S, et al. Deferiprone has less benefits on gut microbiota and metabolites in high iron-diet induced iron overload thalassemic mice than in iron overload wild-type mice: A preclinical study. Life Sci. 2022;307:120871.

5. Quast C, Pruesse E, Yilmaz P, Gerken J, Schweer T, Yarza P, et al. The SILVA ribosomal RNA gene database project: improved data processing and web-based tools. Nucleic Acids Research. 2012;41(D1):D590-D6.

6. Lin H, Peddada SD. Analysis of compositions of microbiomes with bias correction. Nature communications. 2020;11(1):1-11.

7. Yang C, Mai J, Cao X, Burberry A, Cominelli F, Zhang L. ggpicrust2: an R package for PICRUSt2 predicted functional profile analysis and visualization. Bioinformatics. 2023;39(8).

8. Douglas GM, Maffei VJ, Zaneveld JR, Yurgel SN, Brown JR, Taylor CM, et al. PICRUSt2 for prediction of metagenome functions. Nat Biotechnol. 2020;38(6):685-8.

9. Winter SE, Thiennimitr P, Winter MG, Butler BP, Huseby DL, Crawford RW, et al. Gut inflammation provides a respiratory electron acceptor for Salmonella. Nature. 2010;467(7314):426-9.

10. Saiyasit N, Chunchai T, Prus D, Suparan K, Pittayapong P, Apaijai N, et al. Gut dysbiosis develops before metabolic disturbance and cognitive decline in high-fat diet-induced obese condition. Nutrition. 2020;69:110576.

11. Sadaow L, Sanpool O, Rodpai R, Yamasaki H, Ittiprasert W, Mann VH, et al. Development of an Immunochromatographic Point-of-Care Test for Serodiagnosis of Opisthorchiasis and Clonorchiasis. Am J Trop Med Hyg. 2019;101(5):1156-60.

12. Sawaddiruk P, Apaijai N, Paiboonworachat S, Kaewchur T, Kasitanon N, Jaiwongkam T, et al. Coenzyme Q10 supplementation alleviates pain in pregabalin-treated fibromyalgia patients via reducing brain activity and mitochondrial dysfunction. Free Radic Res. 2019;53(8):901-9.

13. Khuankaew C, Apaijai N, Sawaddiruk P, Jaiwongkam T, Kerdphoo S, Pongsiriwet S, et al. Effect of coenzyme Q10 on mitochondrial respiratory proteins in trigeminal neuralgia. Free Radic Res. 2018;52(4):415-25.

14. Thonusin C, IglayReger HB, Soni T, Rothberg AE, Burant CF, Evans CR. Evaluation of intensity drift correction strategies using MetaboDrift, a normalization tool for multi-batch metabolomics data. J Chromatogr A. 2017;1523:265-74.

15. Thonusin C, Nawara W, Khuanjing T, Prathumsup N, Arinno A, Ongnok B, et al. Blood metabolomes as non-invasive biomarkers and targets of metabolic interventions for doxorubicin and trastuzumab-induced cardiotoxicity. Arch Toxicol. 2023;97(2):603-18.

16. Hoving LR, Heijink M, van Harmelen V, van Dijk KW, Giera M. GC-MS Analysis of Short-Chain Fatty Acids in Feces, Cecum Content, and Blood Samples. Methods Mol Biol. 2018;1730:247-56.

Supplementary Table 1. Baseline demographic and clinical characteristics.

Characteristics	CCA (n=56)	HCC (n=14)	p-value
**Demographics**			
Age, year	64.0 (60.0,68.3)	60.5 (52.0,68.0)	0.28
Sex, n (%)			**0.04**
Male	33 (58.9)	12 (85.7)	
Female	23 (41.1)	2 (14.3)	
BW, kg (mean, SD)	55.4 (44.6 - 66.2)	63.9 (51.9 – 75.9)	**0.01**
Risk factor			
Smoking (%)	9 (16.1)	0	0.19
Alcohol consumption (%)	16 (28.6)	3 (27.4)	1.00
Raw food consumption (%)	11 (19.6)	0.0	0.11
NAFLD (%)	0	1 (7.1)	0.19
Cirrhosis (%)	8 (14.3)	10 (71.4)	**<0.01**
HBV infection (%)	2 (3.6)	7 (50.0)	**<0.01**
HCV infection (%)	1 (1.8)	6 (42.9)	**<0.01**
Antibiotics use (%)	2 (3.6)	0	1
Treatment (%)			**<0.01**
Surgery	5 (8.9)	8 (57.1)	
Chemotherapy	48 (85.7)	2 (14.3)	
Multi-kinase inhibitor	0	4 (28.6)	
Supportive care	6 (10.7)	0	
**Tumor staging**			
T (%)			0.31
1-2	39 (69.6)	8 (57.1)	
3-4	17 (30.4)	6 (42.9)	
N (%)			**<0.01**
0	9 (16.1)	13 (92.9)	
1	47 (83.9)	1 (7.1)	
M (%)			**<0.01**
0	10 (17.9)	12 (85.7)	
1	46 (82.1)	2 (14.3)	
Metastatic site (%)			**<0.01**
0-1	36 (64.3)	14 (100.0)	
≥ 2	20 (33.9)	0	

Abbreviations: BW, body weight; CCA, cholangiocarcinoma; HBV, hepatitis B virus; HCC, hepatocellular carcinoma; HCV, hepatitis C virus; kg, kilogram; NAFLD, non-alcoholic fatty liver diseas

Supplementary Table 2. Tissue bacterial profiles using RT-qPCR comparing between ICCA and HCC patients.

Bacterial profiles	ICCA<br>Mean (range)<br>n=56	HCC<br>Mean (range)<br>n=14	Unadjusted mean difference (95 % CI)	p-value	Adjuseted mean difference* (95 % CI)	p-value
Eubacteria (copies/uL, total read count)	102190.5 (0.01–1211055.9)	243694.1 (0.7–1434258.1)	141503.6 (-18383.6 - 301390.8)	0.08	199930.2 (-79723.5 - 479583.8)	0.16
Clostridiales, (%)	50.6 (0.0–1422.5)	53.6 (1.1–356.3)	2.9 (-104.5 - 110.3)	0.96	20.2 (-168.4 - 208.7)	0.83
Lactobacillus, (%)	6.6 (0.0–111.6)	13.7 (0.0–172.8)	7.1 (-9.9 - 24.0)	0.41	1.9 (-27.2 - 31.0)	0.90
Bacteroidota, (%)	192.2 (0.0–10519.3)	25.1 (0.0–346.8)	-167.1 (-921.1 - 586.9)	0.66	47.1 (-1285.3 - 1379.4)	0.94
Enterobacteriaceae, (%)	9.2 (0.0–159.1)	34.34 (0.0–236.11)	25.16 (-1.68 to 51.99)	0.07	-25.1 (-68.7 - 18.6)	0.26
Clostridiales and *Lactobacillus*, (%)	58.16 (0.00–1422.45)	67.22 (1.09–529.11)	9.06 (-103.43 to 121.55)	0.87	22.3 (-174.8 - 219.3)	0.82
F/B ratio	327.58 (0.00–4232.03)	155.18 (0.00–1241.98)	-172.40 (-605.02 to 260.23)	0.43		0.07

Abbreviations: F/B, Firmicutes per Bacteroidota ratio; RT-qPCR, reverse transcriptase-quantitative polymerase chain reaction; 16S rRNA, 16S ribosomal ribonucleic acid.

***Adjusted for cirrhosis status, HBV infection, HCV infection, and metastatic status**

Supplementary Table 3. Tissue bacterial profiles by survival and OV infection status in ICCA patients.

Tissue bacterial profiles	> 6m-PFS (n= 16)	≤ 6m-PFS (n=14)	p-value	> 1yr-OS (n=12)	≤ 1yr-OS (n=18)	p-value	Non-OV (n=21)	OV (n=9)	p-value
Eubacteria (copies/uL)	11410.88 (2883.89, 174963.69)	1887.55 (262.84, 19188.13)	0.23	18049.90 (3274.28, 163937.53)	1896.12 (739.16, 24323.47)	0.37	3585.94 (361.17, 136468.32)	3782.11 (1879.00, 24323.48)	0.92
Clostridiales (%)	3.18 (1.15,5.87)	1.52 (0.89,2.95)	0.21	3.91 (2.03,5.11)	1.47 (0.87,3.02)	1.00	2.45 (1.10,6.01)	1.43 (0.68,2.74)	0.15
Lactobacillus (%)	0.00 (0.00,0.07)	0.01 (0.00,0.29)	0.76	0.02 (0.00,0.18)	0.01 (0.00,0.10)	0.71	0.02 (0.00,0.21)	0.01 (0.00,0.01)	0.48
Bacteroidota (%)	0.07 (0.04,0.26)	0.03 (0.00,0.76)	0.63	0.09 (0.06,0.51)	0.04 (0.00,0.21)	0.24	0.09 (0.04,0.51)	0.01 (0.00,0.07)	0.17
Enterobacteriaceae (%)	0.06 (0.04,0.28)	0.19 (0.03,3.02)	0.43	0.06 (0.05,0.35)	0.10 (0.03,3.00)	0.64	0.101 (0.06,0.57)	0.04 (0.03,0.27)	0.20
Clostridiales and *Lactobacillus* (%)	3.59 (1.15,5.96)	1.80 (0.98,3.11)	0.27	4.56 (2.50,5.22)	1.58 (0.97,3.14)	0.13	2.61 (1.10,7.86)	1.44 (1.01,3.02)	0.24
F/B ratio	49.59 (14.16, 461.14)	94.42 (21.04, 492.84)	0.78	28.83 (14.21, 80.50)	96.31 (18.82, 754.88)	0.40	28.83 (14.21,96.31)	407.47 (70.34, 522.75)	0.31

Abbreviations: F/B, Firmicutes per Bacteroidota ratio; OS, overall survival; OV, Opisthorchis viverini; PFS, progression-free survival.

Supplementary tables 4. Differential abundance of bacterial taxa between two groups

4.1 Comparation between progression of disease on response evaluation by CT scan and no progression

Taxon	FC	SD	p_value	beta_direction	p_significance	color_to_fill_p	tax_level
**Monoglobales**	-2.18	0.72	0.00	Decreased	Sig	down_red	Order
**Clostridiales**	-1.58	0.79	0.05	Decreased	Sig	down_red	Order
**Burkholderiales**	0.75	0.37	0.05	Increased	Sig	up_blue	Order
**Monoglobaceae**	-2.19	0.69	0.00	Decreased	Sig	down_red	Family
**Lactobacillaceae**	-2.06	1.04	0.05	Decreased	Sig	down_red	Family
**[Eubacterium]_coprostanoligenes_group**	-1.76	0.76	0.02	Decreased	Sig	down_red	Family
**Peptostreptococcaceae**	-1.74	0.84	0.04	Decreased	Sig	down_red	Family
**Clostridiaceae**	-1.59	0.79	0.04	Decreased	Sig	down_red	Family
**UCG-010**	-1.50	0.60	0.01	Decreased	Sig	down_red	Family
**Clostridia_vadinBB60_group**	-1.26	0.63	0.04	Decreased	Sig	down_red	Family
**Sutterellaceae**	0.94	0.40	0.02	Increased	Sig	up_blue	Family
**Coriobacteriaceae**	1.72	0.76	0.02	Increased	Sig	up_blue	Family
**Selenomonadaceae**	2.04	1.03	0.05	Increased	Sig	up_blue	Family
**Ruminococcus**	-2.78	0.80	0.00	Decreased	Sig	down_red	Genus
**Monoglobus**	-2.15	0.65	0.00	Decreased	Sig	down_red	Genus
**Romboutsia**	-2.07	0.88	0.02	Decreased	Sig	down_red	Genus
**NK4A214_group**	-2.07	0.74	0.01	Decreased	Sig	down_red	Genus
**Lactobacillus**	-2.02	1.02	0.05	Decreased	Sig	down_red	Genus
**[Ruminococcus]_gauvreauii_group**	-1.82	0.71	0.01	Decreased	Sig	down_red	Genus
**UCG-002**	-1.78	0.81	0.03	Decreased	Sig	down_red	Genus
**Lachnospiraceae_FCS020_group**	-1.77	0.67	0.01	Decreased	Sig	down_red	Genus
**Lachnospiraceae_ND3007_group**	-1.73	0.81	0.03	Decreased	Sig	down_red	Genus
**[Eubacterium]_coprostanoligenes_group**	-1.72	0.68	0.01	Decreased	Sig	down_red	Genus
**Intestinibacter**	-1.70	0.78	0.03	Decreased	Sig	down_red	Genus

**UCG-005**	-1.50	0.74	0.04	Decreased	Sig	down_red	Genus
**Christensenellaceae_R-7_group**	-1.49	0.72	0.04	Decreased	Sig	down_red	Genus
**Lachnospiraceae_UCG-001**	-1.49	0.71	0.04	Decreased	Sig	down_red	Genus
**Intestinimonas**	-1.46	0.45	0.00	Decreased	Sig	down_red	Genus
**UCG-010**	-1.46	0.55	0.01	Decreased	Sig	down_red	Genus
**UCG-003**	-1.44	0.63	0.02	Decreased	Sig	down_red	Genus
**Negativibacillus**	-1.34	0.65	0.04	Decreased	Sig	down_red	Genus
**Paeniclostridium**	-1.32	0.58	0.02	Decreased	Sig	down_red	Genus
**[Eubacterium]_brachy_group**	-1.29	0.63	0.04	Decreased	Sig	down_red	Genus
**d__Bacteria_Actinobacteriota_Coriobacteriia_ Coriobacteriales_Eggerthellaceae_uncultured**	-1.26	0.56	0.03	Decreased	Sig	down_red	Genus
**Clostridia_vadinBB60_group**	-1.22	0.59	0.04	Decreased	Sig	down_red	Genus
**UCG-009**	-0.98	0.45	0.03	Decreased	Sig	down_red	Genus
**Faecalitalea**	-0.95	0.45	0.04	Decreased	Sig	down_red	Genus
**d__Bacteria_Firmicutes_Bacilli_ Erysipelotrichales_Erysipelotrichaceae_uncultured**	-0.84	0.42	0.05	Decreased	Sig	down_red	Genus
**Merdibacter**	-0.78	0.39	0.04	Decreased	Sig	down_red	Genus
**Anaerofilum**	-0.75	0.37	0.04	Decreased	Sig	down_red	Genus
**Sutterella**	1.02	0.40	0.01	Increased	Sig	up_blue	Genus
**Collinsella**	1.76	0.73	0.02	Increased	Sig	up_blue	Genus
**Acidaminococcus**	1.93	0.95	0.04	Increased	Sig	up_blue	Genus

4.2 Comparation between ≤ 6m-PFS compared to > 6m-PFS group

Taxon	FC	SD	p_value	beta_direction	p_significance	color_to_fill_p	tax_level
Desulfuromonadia	-1.60	0.78	0.04	Decreased	Sig	down_red	Class
Clostridiales	-2.09	0.71	0.00	Decreased	Sig	down_red	Order
Monoglobales	-2.08	0.69	0.00	Decreased	Sig	down_red	Order
Christensenellales	-1.56	0.75	0.04	Decreased	Sig	down_red	Order
Clostridia_vadinBB60_group	-1.40	0.65	0.03	Decreased	Sig	down_red	Order
Burkholderiales	0.85	0.35	0.02	Increased	Sig	up_blue	Order
Clostridiaceae	-2.09	0.71	0.00	Decreased	Sig	down_red	Family
Monoglobaceae	-2.08	0.66	0.00	Decreased	Sig	down_red	Family
[Eubacterium]_coprostanoligenes_group	-1.86	0.69	0.01	Decreased	Sig	down_red	Family
UCG-010	-1.70	0.58	0.00	Decreased	Sig	down_red	Family
Christensenellaceae	-1.56	0.71	0.03	Decreased	Sig	down_red	Family
Bradymonadales	-1.45	0.74	0.05	Decreased	Sig	down_red	Family
Clostridia_vadinBB60_group	-1.40	0.62	0.02	Decreased	Sig	down_red	Family
Sutterellaceae	1.08	0.38	0.00	Increased	Sig	up_blue	Family
Ruminococcus	-2.50	0.74	0.00	Decreased	Sig	down_red	Genus
NK4A214_group	-2.33	0.67	0.00	Decreased	Sig	down_red	Genus
Monoglobus	-2.06	0.62	0.00	Decreased	Sig	down_red	Genus
UCG-002	-2.04	0.75	0.01	Decreased	Sig	down_red	Genus
Lachnospiraceae_FCS020_group	-2.02	0.63	0.00	Decreased	Sig	down_red	Genus
Clostridium_sensu_stricto_1	-1.97	0.67	0.00	Decreased	Sig	down_red	Genus
[Eubacterium]_coprostanoligenes_group	-1.84	0.62	0.00	Decreased	Sig	down_red	Genus
Romboutsia	-1.82	0.86	0.04	Decreased	Sig	down_red	Genus
UCG-005	-1.72	0.72	0.02	Decreased	Sig	down_red	Genus
UCG-010	-1.69	0.53	0.00	Decreased	Sig	down_red	Genus
Lachnospiraceae_ND3007_group	-1.67	0.76	0.03	Decreased	Sig	down_red	Genus
Christensenellaceae_R-7_group	-1.62	0.68	0.02	Decreased	Sig	down_red	Genus
Paeniclostridium	-1.61	0.60	0.01	Decreased	Sig	down_red	Genus

Enterobacter	-1.53	0.74	0.04	Decreased	Sig	down_red	Genus
[Ruminococcus]_gauvreauii_group	-1.52	0.69	0.03	Decreased	Sig	down_red	Genus
Intestinimonas	-1.51	0.44	0.00	Decreased	Sig	down_red	Genus
Prevotellaceae_UCG-004	-1.51	0.71	0.03	Decreased	Sig	down_red	Genus
Negativibacillus	-1.49	0.63	0.02	Decreased	Sig	down_red	Genus
d__Bacteria_Actinobacteriota_Coriobacteriia_ Coriobacteriales_Eggerthellaceae_uncultured	-1.48	0.54	0.01	Decreased	Sig	down_red	Genus
Bradymonadales	-1.43	0.71	0.04	Decreased	Sig	down_red	Genus
Family_XIII_AD3011_group	-1.43	0.67	0.03	Decreased	Sig	down_red	Genus
Clostridia_vadinBB60_group	-1.38	0.58	0.02	Decreased	Sig	down_red	Genus
UCG-003	-1.34	0.60	0.02	Decreased	Sig	down_red	Genus
[Eubacterium]_xylanophilum_group	-1.30	0.60	0.03	Decreased	Sig	down_red	Genus
[Eubacterium]_brachy_group	-1.23	0.60	0.04	Decreased	Sig	down_red	Genus
UCG-009	-1.12	0.43	0.01	Decreased	Sig	down_red	Genus
Lactococcus	-1.08	0.54	0.04	Decreased	Sig	down_red	Genus
d__Bacteria_Firmicutes_Bacilli_ Erysipelotrichales_Erysipelotrichaceae_uncultured	-0.95	0.44	0.03	Decreased	Sig	down_red	Genus
Sutterella	1.05	0.40	0.01	Increased	Sig	up_blue	Genus
Acidaminococcus	1.80	0.89	0.04	Increased	Sig	up_blue	Genus

4.3 Comparation between OV-infected patients and non-OV infected patients

Taxon	FC	SD	p_value	beta_direction	p_significance	color_to_fill_p	tax_level
Alphaproteobacteria	-1.80	0.56	0.00	Decreased	Sig	down_red	Class
Monoglobales	-2.43	0.77	0.00	Decreased	Sig	down_red	Order
Clostridiales	-2.18	0.84	0.01	Decreased	Sig	down_red	Order
Rhodospirillales	-1.95	0.54	0.00	Decreased	Sig	down_red	Order
Pasteurellales	-1.55	0.56	0.01	Decreased	Sig	down_red	Order
Micrococcales	-1.40	0.64	0.03	Decreased	Sig	down_red	Order
Clostridia_UCG-014	-1.31	0.65	0.04	Decreased	Sig	down_red	Order
Monoglobaceae	-2.41	0.74	0.00	Decreased	Sig	down_red	Family
Clostridiaceae	-2.16	0.84	0.01	Decreased	Sig	down_red	Family
Leuconostocaceae	-1.97	0.65	0.00	Decreased	Sig	down_red	Family
d__Bacteria_Proteobacteria_Alphaproteobacteria_Rhodospirillales_uncultured	-1.94	0.54	0.00	Decreased	Sig	down_red	Family
Staphylococcaceae	-1.66	0.76	0.03	Decreased	Sig	down_red	Family
Pasteurellaceae	-1.53	0.55	0.01	Decreased	Sig	down_red	Family
Coriobacteriales_Incertae_Sedis	-1.45	0.50	0.00	Decreased	Sig	down_red	Family
Micrococcaceae	-1.37	0.64	0.03	Decreased	Sig	down_red	Family
Clostridia_UCG-014	-1.29	0.63	0.04	Decreased	Sig	down_red	Family
Moraxellaceae	-0.92	0.36	0.01	Decreased	Sig	down_red	Family
Ruminococcus	-2.72	0.88	0.00	Decreased	Sig	down_red	Genus
Enterobacter	-2.57	0.65	0.00	Decreased	Sig	down_red	Genus
Roseburia	-2.54	1.05	0.02	Decreased	Sig	down_red	Genus
Monoglobus	-2.30	0.68	0.00	Decreased	Sig	down_red	Genus
[Eubacterium]_ruminantium_group	-2.29	0.66	0.00	Decreased	Sig	down_red	Genus
Terrisporobacter	-2.20	0.91	0.02	Decreased	Sig	down_red	Genus
Romboutsia	-2.11	0.93	0.02	Decreased	Sig	down_red	Genus
Clostridium_sensu_stricto_1	-1.98	0.79	0.01	Decreased	Sig	down_red	Genus
Weissella	-1.86	0.61	0.00	Decreased	Sig	down_red	Genus
[Eubacterium]_brachy_group	-1.84	0.50	0.00	Decreased	Sig	down_red	Genus

d__Bacteria_Proteobacteria_Alphaproteobacteria_Rhodospirillales_uncultured_uncultured	-1.83	0.55	0.00	Decreased	Sig	down_red	Genus
[Eubacterium]_coprostanoligenes_group	-1.66	0.83	0.05	Decreased	Sig	down_red	Genus
Haemophilus	-1.63	0.49	0.00	Decreased	Sig	down_red	Genus
d__Bacteria_Actinobacteriota_Coriobacteriia_ Coriobacteriales_Coriobacteriales_Incertae_Sedis_uncultured	-1.56	0.44	0.00	Decreased	Sig	down_red	Genus
Staphylococcus	-1.56	0.78	0.04	Decreased	Sig	down_red	Genus
Lactococcus	-1.53	0.52	0.00	Decreased	Sig	down_red	Genus
Sellimonas	-1.51	0.74	0.04	Decreased	Sig	down_red	Genus
Oscillibacter	-1.51	0.54	0.01	Decreased	Sig	down_red	Genus
Negativibacillus	-1.44	0.69	0.04	Decreased	Sig	down_red	Genus
d__Bacteria_Firmicutes_Clostridia_Oscillospirales_Oscillospiraceae_uncultured	-1.32	0.63	0.04	Decreased	Sig	down_red	Genus
Frisingicoccus	-1.30	0.54	0.02	Decreased	Sig	down_red	Genus
Fournierella	-1.24	0.46	0.01	Decreased	Sig	down_red	Genus
Anaerostipes	-1.22	0.56	0.03	Decreased	Sig	down_red	Genus
Intestinimonas	-1.19	0.50	0.02	Decreased	Sig	down_red	Genus
Clostridia_UCG-014	-1.18	0.60	0.05	Decreased	Sig	down_red	Genus
DTU089	-0.82	0.40	0.04	Decreased	Sig	down_red	Genus
Acinetobacter	-0.80	0.38	0.04	Decreased	Sig	down_red	Genus
d__Bacteria_Bacteroidota_Bacteroidia_Bacteroidales_Prevotellaceae_uncultured	2.27	1.07	0.03	Increased	Sig	up_blue	Genus

4.4 Comparation between ≤ 1yr-OS and > 1yr-OS

Taxon	FC	SD	p_value	beta_direction	p_significance	color_to_fill_p	tax_level
Desulfuromonadia	-2.10	0.91	0.02	Decreased	Sig	down_red	Class
Clostridiales	-1.42	0.67	0.03	Decreased	Sig	down_red	Order
Burkholderiales	0.85	0.36	0.02	Increased	Sig	up_blue	Order
Bradymonadales	-1.75	0.88	0.05	Decreased	Sig	down_red	Family
Monoglobaceae	-1.56	0.70	0.03	Decreased	Sig	down_red	Family
Clostridiaceae	-1.54	0.66	0.02	Decreased	Sig	down_red	Family
[Eubacterium]_coprostanoligenes_group	-1.35	0.68	0.05	Decreased	Sig	down_red	Family
Defluviitaleaceae	-1.34	0.55	0.02	Decreased	Sig	down_red	Family
UCG-010	-1.33	0.66	0.04	Decreased	Sig	down_red	Family
Streptococcaceae	-1.02	0.48	0.04	Decreased	Sig	down_red	Family
Sutterellaceae	1.00	0.42	0.02	Increased	Sig	up_blue	Family
Ruminococcus	-2.10	0.67	0.00	Decreased	Sig	down_red	Genus
Bradymonadales	-1.70	0.83	0.04	Decreased	Sig	down_red	Genus
Prevotellaceae_UCG-004	-1.65	0.83	0.05	Decreased	Sig	down_red	Genus
[Eubacterium]_xylanophilum_group	-1.62	0.66	0.01	Decreased	Sig	down_red	Genus
NK4A214_group	-1.58	0.69	0.02	Decreased	Sig	down_red	Genus
Monoglobus	-1.51	0.66	0.02	Decreased	Sig	down_red	Genus
Lachnospiraceae_FCS020_group	-1.45	0.67	0.03	Decreased	Sig	down_red	Genus
Clostridium_sensu_stricto_1	-1.36	0.61	0.02	Decreased	Sig	down_red	Genus
[Eubacterium]_coprostanoligenes_group	-1.30	0.63	0.04	Decreased	Sig	down_red	Genus
Defluviitaleaceae_UCG-011	-1.29	0.55	0.02	Decreased	Sig	down_red	Genus
UCG-010	-1.29	0.59	0.03	Decreased	Sig	down_red	Genus
Intestinimonas	-1.05	0.46	0.02	Decreased	Sig	down_red	Genus
Sutterella	1.01	0.43	0.02	Increased	Sig	up_blue	Genus
Collinsella	1.80	0.83	0.03	Increased	Sig	up_blue	Genus
Acidaminococcus	2.41	0.78	0.00	Increased	Sig	up_blue	Genus

Supplementary tables 5. Gut microbiota correlation with clinical parameters

Feature	taxon	FC	SD	p_value	q_value	beta_direction	p_significance	q_significance	color_to_fill_p	color_to_fill_q	tax_level
hb_1_g_dl	Fusobacteriota	-1.08	0.31	0.00	0.01	Decreased	Sig	Sig	down_red	down_red	Phylum
hb_1_g_dl1	Fusobacteriia	-1.09	0.30	0.00	0.01	Decreased	Sig	Sig	down_red	down_red	Class
hb_1_g_dl2	Fusobacteriales	-1.08	0.30	0.00	0.02	Decreased	Sig	Sig	down_red	down_red	Order
alb_1_g_dl.1	Monoglobales	2.54	0.70	0.00	0.01	Increased	Sig	Sig	up_blue	up_blue	Order
alb_1_g_dl.2	Christensenellales	2.68	0.70	0.00	0.01	Increased	Sig	Sig	up_blue	up_blue	Order
db_1_mg_dl	Bacillales	-0.63	0.16	0.00	0.00	Decreased	Sig	Sig	down_red	down_red	Order
hb_1_g_dl3	Fusobacteriaceae	-1.10	0.31	0.00	0.03	Decreased	Sig	Sig	down_red	down_red	Family
cr_1_mg_dl	Tannerellaceae	-2.33	0.66	0.00	0.03	Decreased	Sig	Sig	down_red	down_red	Family
alb_1_g_dl.14	Monoglobaceae	2.51	0.69	0.00	0.02	Increased	Sig	Sig	up_blue	up_blue	Family
alb_1_g_dl.21	Christensenellaceae	2.66	0.68	0.00	0.01	Increased	Sig	Sig	up_blue	up_blue	Family
tb_1_mg_dl	Coriobacteriales_Incertae_ Sedis	-0.53	0.12	0.00	0.00	Decreased	Sig	Sig	down_red	down_red	Family
db_1_mg_dl.1	Bacillaceae	-0.65	0.16	0.00	0.01	Decreased	Sig	Sig	down_red	down_red	Family
db_1_mg_dl.2	Coriobacteriales_Incertae_ Sedis	-0.56	0.12	0.00	0.00	Decreased	Sig	Sig	down_red	down_red	Family
hb_1_g_dl4	Hungatella	-0.94	0.23	0.00	0.01	Decreased	Sig	Sig	down_red	down_red	Genus
alb_1_g_dl.15	UCG-009	1.70	0.40	0.00	0.01	Increased	Sig	Sig	up_blue	up_blue	Genus
alb_1_g_dl.22	Turicibacter	2.03	0.50	0.00	0.01	Increased	Sig	Sig	up_blue	up_blue	Genus
alb_1_g_dl.3	Family_XIII_UCG-001	2.14	0.38	0.00	0.00	Increased	Sig	Sig	up_blue	up_blue	Genus
alb_1_g_dl.4	Monoglobus	2.49	0.67	0.00	0.04	Increased	Sig	Sig	up_blue	up_blue	Genus
alb_1_g_dl.5	UCG-003	2.84	0.59	0.00	0.00	Increased	Sig	Sig	up_blue	up_blue	Genus
alb_1_g_dl.6	Coprococcus	3.13	0.82	0.00	0.03	Increased	Sig	Sig	up_blue	up_blue	Genus
alb_1_g_dl.7	UCG-005	3.17	0.61	0.00	0.00	Increased	Sig	Sig	up_blue	up_blue	Genus
alb_1_g_dl.8	Christensenellaceae_R-7_group	3.23	0.62	0.00	0.00	Increased	Sig	Sig	up_blue	up_blue	Genus
alb_1_g_dl.9	Lachnospiraceae_FCS020 _group	3.32	0.50	0.00	0.00	Increased	Sig	Sig	up_blue	up_blue	Genus
alb_1_g_dl.10	UCG-002	3.38	0.82	0.00	0.01	Increased	Sig	Sig	up_blue	up_blue	Genus
alb_1_g_dl.11	Family_XIII_AD3011_gro up	3.55	0.65	0.00	0.00	Increased	Sig	Sig	up_blue	up_blue	Genus
alb_1_g_dl.12	Lachnospiraceae_NK4A1 36_group	3.66	0.77	0.00	0.00	Increased	Sig	Sig	up_blue	up_blue	Genus

alb_1_g_dl.13	Dorea	3.74	0.97	0.00	0.03	Increased	Sig	Sig	up_blue	up_blue	Genus
tb_1_mg_dl.1	Turicibacter	-0.51	0.13	0.00	0.03	Decreased	Sig	Sig	down_red	down_red	Genus
tb_1_mg_dl.2	d__Bacteria_Actinobacteriota_Coriobacteriia_ Coriobacteriales_Coriobacteriales_Incertae_Sedis_uncultured	-0.50	0.12	0.00	0.01	Decreased	Sig	Sig	down_red	down_red	Genus
db_1_mg_dl.11	Turicibacter	-0.54	0.14	0.00	0.02	Decreased	Sig	Sig	down_red	down_red	Genus
db_1_mg_dl.21	d__Bacteria_Actinobacteriota_Coriobacteriia_ Coriobacteriales_Coriobacteriales_Incertae_Sedis_uncultured	-0.49	0.12	0.00	0.01	Decreased	Sig	Sig	down_red	down_red	Genus
Acetic	Actinomycetales	-0.13	0.04	0.00	0.01	Decreased	Sig	Sig	down_red	down_red	Order
Isobutyric.1	Peptostreptococcales-Tissierellales	1.32	0.38	0.00	0.02	Increased	Sig	Sig	up_blue	up_blue	Order
Isobutyric.2	Clostridiales	2.51	0.66	0.00	0.01	Increased	Sig	Sig	up_blue	up_blue	Order
Isovaleric.1	Lactobacillales	-1.18	0.35	0.00	0.03	Decreased	Sig	Sig	down_red	down_red	Order
Isovaleric.2	Peptostreptococcales-Tissierellales	1.38	0.42	0.00	0.05	Increased	Sig	Sig	up_blue	up_blue	Order
Isovaleric.3	Clostridiales	2.54	0.70	0.00	0.01	Increased	Sig	Sig	up_blue	up_blue	Order
Acetic1	Actinomycetaceae	-0.13	0.04	0.00	0.02	Decreased	Sig	Sig	down_red	down_red	Family
Isobutyric.110	Anaerovoracaceae	1.48	0.36	0.00	0.00	Increased	Sig	Sig	up_blue	up_blue	Family
Isobutyric.21	Oscillospiraceae	1.68	0.38	0.00	0.00	Increased	Sig	Sig	up_blue	up_blue	Family
Isobutyric.3	Clostridiaceae	2.47	0.66	0.00	0.01	Increased	Sig	Sig	up_blue	up_blue	Family
Isovaleric.16	Anaerovoracaceae	1.43	0.38	0.00	0.01	Increased	Sig	Sig	up_blue	up_blue	Family
Isovaleric.21	Oscillospiraceae	1.83	0.46	0.00	0.01	Increased	Sig	Sig	up_blue	up_blue	Family
Isovaleric.31	Clostridiaceae	2.51	0.69	0.00	0.02	Increased	Sig	Sig	up_blue	up_blue	Family
Isobutyric.111	Unknown	0.95	0.24	0.00	0.01	Increased	Sig	Sig	up_blue	up_blue	Genus
Isobutyric.22	Lachnospiraceae_NC2004_group	1.07	0.26	0.00	0.01	Increased	Sig	Sig	up_blue	up_blue	Genus
Isobutyric.31	UCG-009	1.39	0.35	0.00	0.01	Increased	Sig	Sig	up_blue	up_blue	Genus
Isobutyric.4	Christensenella	1.42	0.34	0.00	0.01	Increased	Sig	Sig	up_blue	up_blue	Genus
Isobutyric.5	Intestinimonas	1.60	0.39	0.00	0.01	Increased	Sig	Sig	up_blue	up_blue	Genus
Isobutyric.6	d__Bacteria_Firmicutes_Clostridia_Oscillospirales_	1.83	0.38	0.00	0.00	Increased	Sig	Sig	up_blue	up_blue	Genus

	Ruminococcaceae_uncultured										
Isobutyric.7	Anaerofilum	1.87	0.25	0.00	0.00	Increased	Sig	Sig	up_blue	up_blue	Genus
Isobutyric.8	Lachnospiraceae_FCS020_group	1.93	0.50	0.00	0.03	Increased	Sig	Sig	up_blue	up_blue	Genus
Isobutyric.9	Family_XIII_UCG-001	1.93	0.45	0.00	0.00	Increased	Sig	Sig	up_blue	up_blue	Genus
Isobutyric.10	Phocea	2.01	0.33	0.00	0.00	Increased	Sig	Sig	up_blue	up_blue	Genus
Isobutyric.11	DTU089	2.02	0.52	0.00	0.02	Increased	Sig	Sig	up_blue	up_blue	Genus
Isobutyric.12	Oscillibacter	2.06	0.51	0.00	0.01	Increased	Sig	Sig	up_blue	up_blue	Genus
Isobutyric.13	d__Bacteria_Firmicutes_Clostridia_Oscillospirales_Oscillospiraceae_uncultured	2.10	0.39	0.00	0.00	Increased	Sig	Sig	up_blue	up_blue	Genus
Isobutyric.14	Negativibacillus	2.27	0.49	0.00	0.00	Increased	Sig	Sig	up_blue	up_blue	Genus
Isobutyric.15	Romboutsia	2.34	0.63	0.00	0.05	Increased	Sig	Sig	up_blue	up_blue	Genus
Isobutyric.16	d__Bacteria_Actinobacteriota_Coriobacteriia_Coriobacteriales_Atopobiaceae_uncultured	2.42	0.52	0.00	0.00	Increased	Sig	Sig	up_blue	up_blue	Genus
Isobutyric.17	UCG-002	2.46	0.56	0.00	0.00	Increased	Sig	Sig	up_blue	up_blue	Genus
Isobutyric.18	Clostridium_sensu_stricto_1	2.57	0.61	0.00	0.01	Increased	Sig	Sig	up_blue	up_blue	Genus
Isobutyric.19	UCG-005	2.78	0.55	0.00	0.00	Increased	Sig	Sig	up_blue	up_blue	Genus
Isobutyric.20	Family_XIII_AD3011_group	2.94	0.46	0.00	0.00	Increased	Sig	Sig	up_blue	up_blue	Genus
Isovaleric.17	Lachnospiraceae_NC2004_group	1.02	0.27	0.00	0.04	Increased	Sig	Sig	up_blue	up_blue	Genus
Isovaleric.22	UCG-009	1.42	0.38	0.00	0.04	Increased	Sig	Sig	up_blue	up_blue	Genus
Isovaleric.32	Christensenella	1.46	0.34	0.00	0.00	Increased	Sig	Sig	up_blue	up_blue	Genus
Isovaleric.4	Anaerofilum	1.79	0.30	0.00	0.00	Increased	Sig	Sig	up_blue	up_blue	Genus
Isovaleric.5	d__Bacteria_Firmicutes_Clostridia_Oscillospirales_Ruminococcaceae_uncultured	1.83	0.44	0.00	0.01	Increased	Sig	Sig	up_blue	up_blue	Genus
Isovaleric.6	Family_XIII_UCG-001	1.90	0.49	0.00	0.03	Increased	Sig	Sig	up_blue	up_blue	Genus
Isovaleric.7	Phocea	2.02	0.37	0.00	0.00	Increased	Sig	Sig	up_blue	up_blue	Genus
Isovaleric.8	Oscillibacter	2.06	0.54	0.00	0.03	Increased	Sig	Sig	up_blue	up_blue	Genus
Isovaleric.9	d__Bacteria_Firmicutes_Clostridia_Oscillospirales_	2.13	0.45	0.00	0.00	Increased	Sig	Sig	up_blue	up_blue	Genus

	Oscillospiraceae_uncultured										
Isovaleric.10	Negativibacillus	2.31	0.52	0.00	0.00	Increased	Sig	Sig	up_blue	up_blue	Genus
Isovaleric.11	UCG-002	2.43	0.62	0.00	0.02	Increased	Sig	Sig	up_blue	up_blue	Genus
Isovaleric.12	d__Bacteria_Actinobacteriota_Coriobacteriia_ Coriobacteriales_Atopobiaceae_uncultured	2.51	0.52	0.00	0.00	Increased	Sig	Sig	up_blue	up_blue	Genus
Isovaleric.13	Clostridium_sensu_stricto_1	2.60	0.65	0.00	0.01	Increased	Sig	Sig	up_blue	up_blue	Genus
Isovaleric.14	UCG-005	2.80	0.56	0.00	0.00	Increased	Sig	Sig	up_blue	up_blue	Genus
Isovaleric.15	Family_XIII_AD3011_group	2.92	0.49	0.00	0.00	Increased	Sig	Sig	up_blue	up_blue	Genus
Valeric	Anaerofilum	1.05	0.21	0.00	0.00	Increased	Sig	Sig	up_blue	up_blue	Genus

Supplementary tables 6. Shared predictive metabolite pathway and clinical outcomes

**PD (n=12)**	PD and Death (n=0)	PD, 6mpd and Death (n=0)	PD and 6mpd (n=7)	PD and OV (n=4)	PD, OV and Death (n=0)	PD, 6mpd, OV, and Death (n=0)	PD, 6mpd and OV (n=0)	OV (n=8)	OV and Death (n=0)	6mpd, OV and Death (n=0)	6mpd and OV (n=0)	Death (n=3)	6mpd and Death (n=2)	6mpd (n=4)
superpathway of L-arginine, putrescine, and 4-aminobutanoate degradation	not found	not found	erythromycin D biosynthesis	phospholipases	not found	not found	not found	Mandelate degradation I	not found	not found	not found	mixed acid fermentation	toluene degradation VI (anaerobic)	glycine betaine degradation I
**superpathway of L-arginine and L-ornithine degradation**			superpathway of 2,3-butanediol biosynthesis	mandelate degradation to acetyl-CoA				mycolyl-arabinogalactan-peptidoglycan complex biosynthesis				ppGpp bio-synthesis	androstene dione degradation	myo-inositol degradation I
**D-galactarate degradation I**			6-hydroxymethyl-dihydropterin diphosphate biosynthesis III (Chlamydia)	superpathway of aerobic toluene degradation				4-hydroxyphenylacetate degradation				tRNA processing		paromamine biosynthesis II
**superpathway of D-glucarate and D-galactarate degradation**			glycerol degradation to butanol	ethylmalonyl-CoA pathway				toluene degradation III (aerobic) (via p-cresol)						coenzyme M biosynthesis I
**NAD salvage pathway II**			6-hydroxymethyl-dihydropterin diphosphate biosynthesis I					mannan degradation						
**aerobactin biosynthesis**			D-glucarate degradation I					L-methionine salvage cycle I (bacteria and plants)						
**superpathway of ornithine degradation**			androstenedione degradation					toluene degradation I (aerobic) (via o-cresol)						
**enterobacterial common antigen biosynthesis**								toluene degradation II (aerobic) (via 4-methylcatechol)						
**creatinine degradation II**														
**peptidoglycan biosynthesis II (staphylococci)**														
**sulfoglycolysis**														
**enterobactin biosynthesis**														

Supplementary Table 7. Univariate analysis of prognostic factors associated with poor outcomes.

Characteristics	Response evaluation	6m-PFS	1y-OS	OV
	p-value	p-value	p-value	p-value
Age	0.65	0.35	0.85	0.17
Sex	0.24	0.07	0.08	0.34
BW	0.74	1.00	0.51	0.64
Height	0.37	0.24	0.33	0.29
Smoking	0.49	0.28	0.30	0.93
Alcohol consumption	0.87	0.83	0.87	0.59
Raw food consumption	0.15	0.28	0.71	0.44
Cirrhosis	0.34	0.48	0.80	0.99
HBV infection	0.77	0.92	0.77	0.53
HCV infection	0.99	1.00	0.99	1.00
Antibiotics use	0.99	0.99	1.00	0.99
OV	0.06	***0.04***	0.21	1.00
Staging T	0.29	0.90	0.95	0.57
Staging N	0.80	0.48	0.80	0.89
Staging M	0.52	0.37	0.66	0.82
Hb	0.76	0.71	0.71	0.18
WBC	0.60	0.86	0.86	0.10
ANC	0.56	0.73	0.77	0.08
PMN	0.60	0.55	0.19	0.30
ALC	0.18	0.11	***0.02***	0.55
Lymphocyte	0.06	0.09	***0.03***	0.36
AEC	0.06	0.10	0.14	0.78
Eosinophils	***0.04***	0.06	0.11	0.52
Platelets	0.46	0.34	0.33	0.11
N/L ratio	0.23	0.11	0.07	0.22
P/L ratio	0.33	0.12	***0.05***	0.39
Creatinine	***0.03***	0.07	0.38	0.57
Alb	0.09	0.09	0.41	0.40
Glb	0.73	0.97	0.30	0.72
ALP	0.37	0.54	0.51	0.55
AST	0.30	0.44	0.39	1.00
ALT	0.49	0.50	0.39	0.60
TB	0.89	0.94	0.69	0.65
DB	0.85	0.92	0.56	0.64
CEA	0.42	0.58	0.33	0.41
CA 19-9	0.54	0.49	0.38	0.48
Log_{10} CA 19-9	0.51	0.84	0.59	0.29
AFP	1.00	1.00	0.75	0.70
Oxidative stress	0.61	0.65	0.83	0.37
IL-10	0.61	0.51	0.55	0.63
IL-1B	0.43	0.37	0.56	0.61

Characteristics	Response evaluation	6m-PFS	1y-OS	OV
	p-value	p-value	p-value	p-value
IL-6	0.56	0.51	0.55	0.63
MCP-1	0.75	0.51	0.64	0.42
TNF-alpha	0.67	0.47	0.56	0.63
Chenodeoxycholic acid	0.49	0.51	0.78	0.15
Cholic acid	0.71	0.53	0.66	0.19
Glycocholic acid	0.37	0.44	0.79	0.66
Glycodeoxycholic acid and glycoursodeoxycholic acid	0.28	0.30	0.38	0.99
Taurochenodeoxycholic acid and tauroursodeoxycholic acid	0.06	0.12	0.15	0.62
Taurocholic acid	0.50	0.50	0.54	0.64
Acetic acid	0.76	0.37	0.51	0.11
Propionic acid	0.70	0.55	0.87	0.11
Isobutyric acid	0.25	0.41	0.85	0.28
Butyric acid	0.54	0.76	0.71	0.14
Isovaleric acid	0.31	0.51	0.98	0.35
Valeric acid	0.30	0.86	0.86	0.28
Acidaminococcus	0.10	0.18	0.13	0.31
Sutterella	0.07	0.13	0.13	0.29
NK4A214_*group*	0.93	0.68	0.55	0.82
Lachnospiraceae_FCS020_group	0.09	***0.04***	0.07	0.36
UCG-010	0.23	0.14	0.25	0.26
Ruminococcus	0.09	0.05	***0.05***	0.09
Monoglobus	0.06	***0.03***	***0.03***	0.18
(25)_coprostanoligenes_group	0.07	***0.02***	0.17	0.41
Intestinimonas	***0.05***	***0.03***	0.35	0.28

Abbreviations: AFP, alpha-fetoprotein Alb, albumin; ALP, alkaline phosphatase; AST, *aspartate aminotransferase*; ALT, alanine transaminase; AEC, absolute eosinophil count; ALC, absolute lymphocyte count; ANC, absolute neutrophic count; BW, body weight; CA 19-9, cancer antigen 19-9; CEA, carcinoembryonic antigen; DB, direct bilirubin; Glb, globulin; Hb, hemoglobin; HBV, hepatitis B virus; HCV, hepatitis C virus; ICCA, intrahepatic cholangiocarcinoma; IL, interleukin; MCP-1, *monocyte chemoattractant protein-1*, N/L, neutrophil per lymphocyte; OS, overall survival; OV, *Opisthorchis viverini*; PFS, progression-free survival; P/L, platelet per lymphocyte; TB, total bilirubin; TNF, tumor necrotic factor; WBC, white blood count.
